# Prefronto-cortical dopamine D1 receptor sensitivity can critically influence working memory maintenance during delayed response tasks

**DOI:** 10.1371/journal.pone.0198136

**Published:** 2018-05-29

**Authors:** Melissa Reneaux, Rahul Gupta

**Affiliations:** School of Computational and Integrative Sciences, Jawaharlal Nehru University, New Delhi, India; Universidad de Salamanca, SPAIN

## Abstract

The dopamine (DA) hypothesis of cognitive deficits suggests that too low or too high extracellular DA concentration in the prefrontal cortex (PFC) can severely impair the working memory (WM) maintenance during delay period. Thus, there exists only an optimal range of DA where the sustained-firing activity, the neural correlate of WM maintenance, in the cortex possesses optimal firing frequency as well as robustness against noisy distractions. Empirical evidences demonstrate changes even in the D1 receptor (D1R)-sensitivity to extracellular DA, collectively manifested through D1R density and DA-binding affinity, in the PFC under neuropsychiatric conditions such as ageing and schizophrenia. However, the impact of alterations in the cortical D1R-sensitivity on WM maintenance has yet remained poorly addressed. Using a quantitative neural mass model of the prefronto-mesoprefrontal system, the present study reveals that higher D1R-sensitivity may not only effectuate shrunk optimal DA range but also shift of the range to lower concentrations. Moreover, higher sensitivity may significantly reduce the WM-robustness even within the optimal DA range and exacerbates the decline at abnormal DA levels. These findings project important clinical implications, such as dosage precision and variability of DA-correcting drugs across patients, and failure in acquiring healthy WM maintenance even under drug-controlled normal cortical DA levels.

## Introduction

Working memory (WM) is a crucial asset of cognitive facility during delayed-response tasks. It is comprised of many subprocesses, namely, attentional control system, retention of cue-induced information over a brief delay interval (WM maintenance), and other executive functions performing manipulation as well as retrieval of cue-specific information at the end of the delay period. These processes concertedly guide the goal-directed response. However, WM maintenance lies at the core of these various cognitive operations [1]. Sustained/persistent-firing activity in the cortices of human as well as non-human primate brains during delay is the proposed neural correlate of WM maintenance [2]. Although participation of various regions of the cortex, including prefrontal cortex (PFC), posterior parietal cortex (PPC) and inferior temporal cortex (ITC), has been observed in WM maintenance [3], the PFC is known to play a pivotal role.

The neurochemical dopamine (DA) exerts a strong modulating effect on WM. Although the effect of DA is mediated through the activation of D1 receptors (D1Rs) as well as D2 receptors (D2Rs) present locally in the cortical region, it is suggested that the effect on WM maintenance is predominantly mediated through the activation of D1Rs whereas D2Rs are primarily involved in the WM updating and executive functions [4, 5]. The computational studies [6–10] and experimental studies [11–15] have brought immense growth in our understanding of the dopaminergic modulation of WM maintenance. These attempts have led to the well-known DA hypothesis of cognitive deficit observed under various neuropsychiatric conditions, such as ageing [16, 17], stress [18, 19], and schizophrenia [15, 20]. According to this hypothesis, too low or too high extracellular DA concentration in the PFC can severely impair the WM maintenance during delay period. Thus, there exists only an optimal range of DA where the WM-associated sustained-firing activity in the cortex possesses optimal firing frequency as well as robustness against noisy distractions.

However, several experimental studies [16, 17, 21–27] have also reported alterations even in the cortical D1R density and reactivity of DA-binding sites on individual D1Rs under various neuropsychiatric conditions. Together, these factors critically regulate the efficiency of the local cortical network for detecting changes in the extracellular DA content and, thus, define the D1R-sensitivity of the cortical region. The D1R-sensitivity is experimentally measured in terms of binding potential (BP) of D1Rs in the PFC [17, 23, 26]. Accordingly, the alteration in D1R-sensitivity appears as an additional important factor to be considered in conjunction with the alteration in cortical DA content. However, the impact of alteration in D1R-sensitivity on the WM maintenance has still remained unaddressed.

The present study addresses this issue by employing a quantitative neural mass model of the prefronto-mesoprefrontal system, which is comprised of the reciprocal interaction between the PFC and the cortical-projecting DA neurons residing in the ventral tegmental area (VTA) in the midbrain [28]. Particularly, the effects of D1R-sensitivity on the firing frequency and robustness of the cortical persistent activity during delay are observed. Moreover, the mesocortical scale of the framework facilitates quantitative observation on the variation in modulation-associated extracellular DA under different conditions of the sensitivity. The findings suggest that cortical D1R-sensitivity critically governs the range of cortical DA level underlying the modulation of WM maintenance in the physiological scenario. Interestingly, this regulation is a consequence of the feedback control of cortical D1R-sensitivity on the dynamics of DA release from VTA-residing DA neurons during delay. Accordingly, increase in D1R-sensitivity causes shrinking of the optimal DA range and shift of the range to lower concentrations. This essentially curtails the safe DA range of efficient WM maintenance in the PFC in the presence of physiological fluctuations in the cortical DA. Furthermore, besides exacerbating the decline in WM-robustness at abnormal DA levels, increased sensitivity is characterized with lesser robustness of the persistent cortical activity even within the optimal DA range.

## Methods

**Fig 1:**
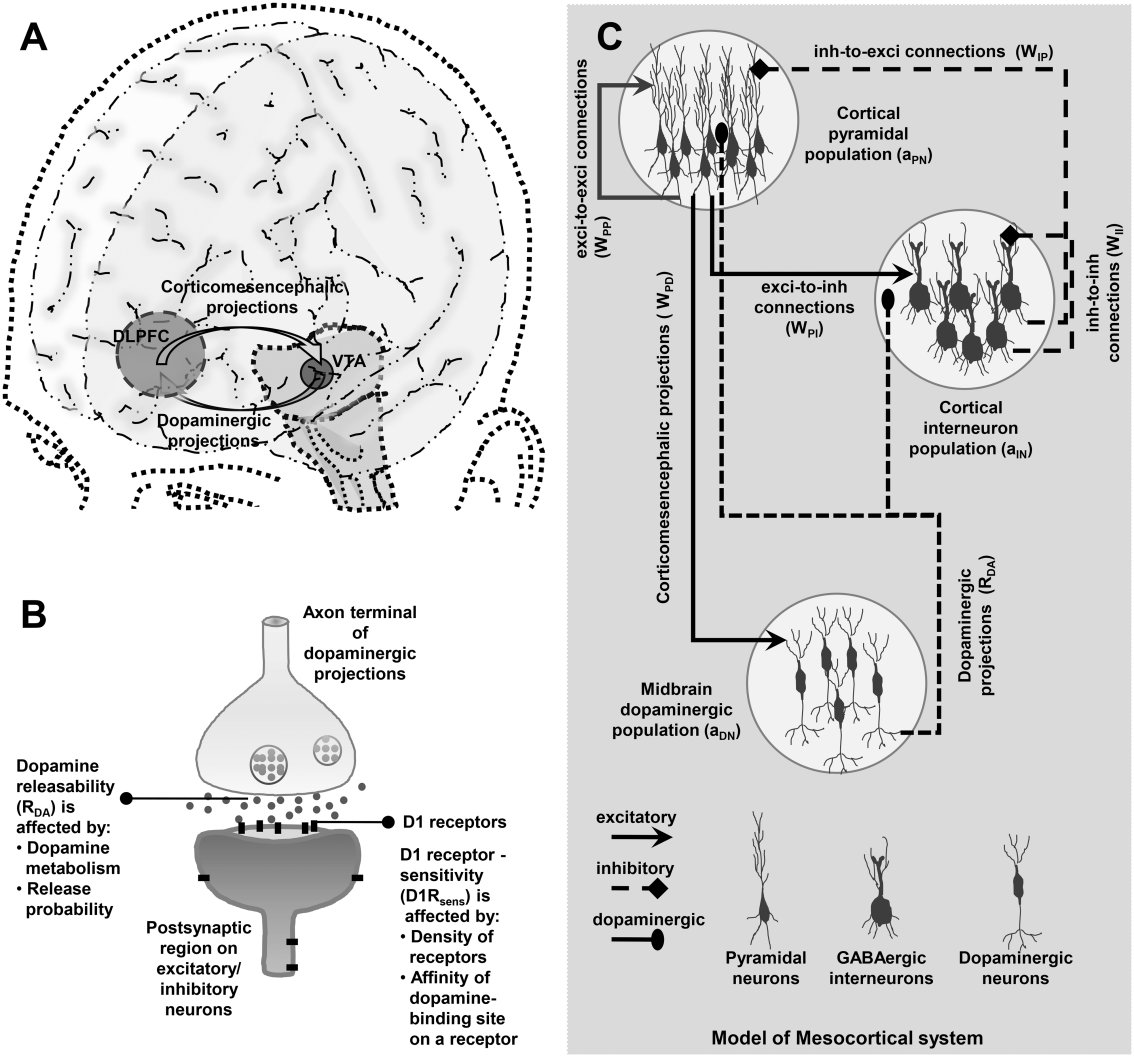
Model of the closed-loop mesocortical circuit. (A) A three-dimensional minimal rendering of the human brain essentially featuring the anatomical localization of the two brain regions, DLPFC and VTA, whose reciprocal interaction constitutes the mesocortical circuit. (B) A simplified illustration of the synaptic contact made by a terminal of the dopaminergic afferent projections onto a pyramidal neuron or GABAergic interneuron in the cortex. The DA-releasability (*R*_*DA*_) and D1R-sensitivity (*D*1*R*_*sens*_) are the presynaptic and postsynaptic factors, respectively, which crucially regulate the transmission at dopaminergic synapses. (C) In the neural mass model of the mesocortical circuit, the cortical neurons are broadly categorized into the populations of excitatory pyramidal neurons and inhibitory GABAergic interneurons. The excitatory population, on receiving cue input, self excites itself (with the synaptic efficacy *W*_*PP*_) and also excites the population of inhibitory neurons in the cortex (*W*_*PI*_) as well as DA neurons in midbrain (*W*_*PD*_). On excitation, the inhibitory population inhibits excitatory population (*W*_*IP*_) as well as itself (*W*_*II*_) whereas the DA neuron population releases DA in the cortex (*R*_*DA*_) through dopaminergic projections and causes accumulation of the cortical DA pool, [*DA*].

The particular subset of the larger prefronto-mesoprefrontal system modeled here includes interactions between a local population of cortical neurons in the dorsolateral prefrontal cortex (DLPFC) extending corticomesencephalic glutamatergic projections [29, 30] to a subpopulation of DA neurons in the VTA, which in turn sends mesocortical dopaminergic projections [31] to the cortical population. In this way, the reciprocal interaction (Fig 1A) gives rise to the mesocortical circuit. The DLPFC is a cortical region within the PFC and has been observed to be actively involved in many visuospatial WM tasks [32–34]. The mathematical model [35] employed here adopts a neural mass approach where the population-averaged activities of the different kinds of neuronal populations constituting the circuit dynamics are considered. The present model provides quantitative profiles of the various measurable entities of the mesocortical dynamics in close association with their experimentally known estimates. Further, a stochastic formulation of the mass model [36] is utilized to gain features of robustness of the WM maintenance during delay under the physiologically-relevant situation of noisy mesocortical dynamics.

### Modeling the dynamics of local cortical network

Glutamate-releasing excitatory pyramidal neurons and GABA-releasing inhibitory interneurons are the most abundant neurons in the PFC. The layer V-VI (deep-layer) neurons are the subject of interest here as they have been found, to be mainly associated with the recurrent sustained firing activity during WM-tasks [37]. The superficial layers are mainly involved in receiving afferent stimuli from various parts of the brain, such as thalamus and intercortical regions, and transmit them to the deep layers. Delayed-response tasks, such as spatial tasks, have demonstrated different cortical neurons to be specifically tuned to firing in response to a characteristic stimuli presented [38, 39]. Therefore, there exists local clusters of cortical neurons which fire maximally towards a specific external stimuli, such as orientation in space in the spatial tasks, than the others.

Under the present neural mass framework, the excitatory and inhibitory neurons in a local cortical network are pooled into distinct populations and the interactions among them are considered at the population-level. Accordingly, DLPFC activity is comprised of the local population activity of excitatory pyramidal neurons (*a*_*PN*_). The pyramidal population self-excites itself with the synaptic efficacy *W*_*PP*_ (feed-forward excitation) and excites the population of local GABAergic interneurons with the synaptic efficacy *W*_*PI*_. In turn, the activity of interneuron population (*a*_*IN*_) exerts inhibition on *a*_*PN*_ with the synaptic efficacy *W*_*IP*_ (feed-back inhibition) as well as suppresses itself with the synaptic efficacy *W*_*II*_. This interplay between the feed-forward excitation and the feed-back inhibition leads to the establishment of sustained-firing activity in the DLPFC, which represents the formation and maintenance of WM during delay period.

$$\begin{array}{c} \frac{da_{PN}(t)}{dt}=-\frac{\Delta a_{PN}(t)}{\tau_{PN}}+W_{PP}f(c_{1}\Delta a_{PN})-W_{IP}f(c_{2}\Delta a_{IN}) \end{array}$$ (1)

$$\begin{array}{c} \frac{da_{IN}(t)}{dt}=-\frac{\Delta a_{IN}(t)}{\tau_{IN}}+W_{PI}f(c_{1}\Delta a_{PN})-W_{II}f(c_{2}\Delta a_{IN}) \end{array}$$ (2)

where, $\Delta a_{PN}(t)=a_{PN}(t)-a_{PN}^{basal}$ and $\Delta a_{IN}(t)=a_{IN}(t)-a_{IN}^{basal}$. The $a_{PN}^{basal}$ and $a_{IN}^{basal}$ corresponds to the basal spontaneous activity level in the pyramidal and GABAergic interneuron populations, respectively, in the local cortical network in the PFC.

The activation function, *f*(Δ*x*) where Δ*x*(*t*) ∈ {Δ*a*_*PN*_(*t*), Δ*a*_*IN*_(*t*), Δ*a*_*DN*_(*t*), Δ[*DA*](*t*)} signifies a biophysically-imposed finite saturating limit to which the different variables may rise during their activation and is given by,

$$\begin{array}{c} f(\Delta x)=\{\begin{array}{c} \tanh(C\Delta x),\Delta x(t)\geq 0\\ 0,\Delta x(t)<0 \end{array}\} \end{array}$$ (3)

here, *C* denotes the constants *c*_1_, *c*_2_, *c*_3_, *c*_4_ associated with the *tanh* function of Δ*a*_*PN*_(*t*), Δ*a*_*IN*_(*t*), Δ*a*_*DN*_(*t*), Δ[*DA*](*t*), respectively.

The first term on the right-hand side of Eqs 1 and 2 denotes the excitability of the population of pyramidal neurons and interneurons characterized by the specific time constants *τ*_*PN*_ and *τ*_*IN*_, respectively. A large time constant implies a greater excitability of the neurons constituting a population. The second term in Eqs 1 and 2 represents the recurrent excitation of the pyramidal neurons and excitation of interneurons by the pyramidal activity, respectively, with the corresponding synaptic efficacies *W*_*PP*_ and *W*_*PI*_. The last term in these equations represents the inhibition of pyramidal population by interneuron population and self-inhibition of interneuron population, respectively, with the corresponding synaptic efficacies *W*_*IP*_ and *W*_*II*_.

### Modeling the dynamics of cortical DA regulation

According to the standing literature, there still exist numerous elements of confusion regarding the regulation of cortical DA during WM maintenance. Particularly, a definitive conclusion could not be drawn yet regarding the pertinent roles of tonic vs. phasic release of DA in the cortex as well as the associated tonic and phasic activities of the cortical-projecting sub-population of DA neurons residing in the midbrain region.

In vivo single-cell recordings of midbrain dopamine (DA) neurons in monkeys have demonstrated a continuous occurrence of basal-level spontaneous activity in the DA neurons [40, 41]. This tonic activity of DA neurons leads to the tonic release of DA at various target sites in the brain innervated by dopaminergic projections [42]. Accordingly, the tonic activity of cortical-projecting DA neurons in the VTA serves as the source for the stable and volume-wide basal extracellular DA concentration in the PFC. In addition, there also occurs heightened phasic burst-activity in the DA neurons mainly at two instances during delayed-response tasks [40, 41]. First, it occurs at the initial instance of cue presentation and serves to alert subject’s attention towards the external stimuli of salience for correctly performing the task. Secondly, it occurs at the eventual moment of making a motor response in the expectation of reward. These evoked phasic activities lead to a sudden excessive release of DA from the afferent dopaminergic terminals at the target sites [42–44]. However, synaptic as well as extrasynaptic rapid uptake of DA by the local dopamine uptake transporters (DATs) and catechol-O-methyl transferase (COMT)-based degradation of DA lead to only a transient high amplitude pulse-like increase in DA concentration in very close vicinity of the release sites [42, 44]. This manner of DA release is commonly referred to as the phasic mode of DA release [42]. Accordingly, phasic DA release does not considerably affect the extracellular DA concentration across a wide volume [42, 44].

Interestingly, sustained activity in DA neurons has not been observed during delay periods in primates VTA undergoing delayed alternation tasks [40]. Although later experiments revealed increase in DA activity during delay [41], this modulation in DA activity was mainly attributed to the intensity of reward probability and uncertainty, rather than to the sustained-firing activity in the PFC. Accordingly, it is implied that the tonic activity of the VTA-residing DA neurons does not change during the delay interval. It has been often suggested that the phasic cortical DA release at the instance of cue presentation may underlie the dopaminergic modulation of WM maintenance during delay. This possibility immediately connects to the gating hypothesis of the dopaminergic modulation [45, 46]. It suggests that phasic activity of the DA neurons at cue presentation initially gates the input stimuli associated with the WM updation and later facilitates WM maintenance by restraining the entry of distracting stimuli [12] during delay. Although the phasic DA release is transient and its influence is spatially-restricted within close vicinity of the DA release sites, the slow intracellular DA signaling [13] and the presence of statistically-significant population of dopaminergic synapses closely-apposed to the asymmetric excitatory synapses in the cortical region [47] may strongly support the gating hypothesis.

However, besides gating of the input, various experimental [11, 13, 18] and theoretical studies [6, 7, 9, 10] have also suggested dopaminergic modulation of the intrinsic excitability and robustness of the cortical neural networks to distracting stimuli during delay intervals, which is also the prime objective of the present modeling study. This would require a volume-wide stable change in cortical DA, as D1Rs are mostly located extrasynaptic to the site of DA release [48]. In fact, in the behaving rhesus monkeys correctly performing in the delayed alternation tasks, a noticeable increase over the basal DA level in the DLPFC has been reported through in vivo microdialysis [49]. Such a change is certainly beyond the capacity of phasic DA release. Although the sparse presence of DATs in the prefrontal cortex [50–52] had been doubted to enable phasic DA release for causing a volume-wide change in DA level, the experimental observations on the sparse DA projections as well as DA release sites [53] in the PFC relative to the striatum and the uptake of DA by the norepinephrine uptake transporters present in high density on the local norepinephrinergic afferent projections [54–56] again seem to decline such a possibility. In fact, a recent detailed computational study by Spühler and Hauri [57] of the spatiotemporal features of DA release in macaque prefrontal cortex has also demonstrated lack of a volume-wide stable change in cortical DA level due to the phasic activities in cortical-projecting DA neurons.

These observations suggest that the involvement of tonic DA release is indispensable to the volume-wide modulation of network excitability and robustness during delay. In the context of striatum, Grace [58] has proposed a plausible mechanism for the change in the local tonic DA release without any change in the tonic activity of the striatum-projecting DA neurons. It suggests that local activity-dependent change in the extracellular glutamate concentration can regulate the tonic DA release through ionotropic AMPA and NMDA receptors located at the afferent dopaminergic terminals. However, the electron microscopic investigations [59, 60] of the distribution of immunofluorescently-labelled ionotropic glutamate receptors in the rodent striatum did not demonstrate a statistically-significant presence of these receptors on the dopaminergic terminals. Further, it has been experimentally observed that there occurs increase in extracellular DA concentration but no change in the extracellular glutamate concentration in the DLPFC during delayed alternation tasks performed by healthy rhesus monkeys [61].

Eventually, it appears that delay-associated change in the tonic activity of cortical-projecting DA neurons may underlie the change in tonic DA release in the cortex. In fact, application of NMDA and AMPA agonist (antagonist) in rodent VTA has been shown to cause increase (decrease) in the extracellular DA level in the PFC [62]. Therefore, change in the local glutamate concentration in the VTA through VTA-projecting cortical neurons may influence the tonic activity of DA neurons during delay. However, it also demands a reconsideration of the abovementioned experimental observations on the lack of sustained activity in the midbrain DA neurons during delay. In this regard, it must be noted that the DA neurons recorded during delayed tasks in these studies [40, 41] were not specific to cortical-projecting DA neuron sub-population in the VTA. Rather, DA neurons belonging to a wide range of projection areas were collectively sampled in the VTA as well as the substantia nigra pars compacta. In contrast, the closed-loop mesocortical circuit addressed here involves the specific DA neuron sub-population which receives excitatory signals from the PFC as well as project back to cortical region.

Moreover, not all the mesocortical DA neurons in the VTA fire under basal resting conditions [42]. Rather, a significant proportion of these neurons remain in the hyperpolarized inactive state. However, during delay period of WM tasks, the increased glutamate level in the VTA due to sustained activity in the PFC may lead to activation of more fractions of inactive DA neurons. It is thought that early activation of DA neurons from their inactive state leads to tonic mode of Poissonian firing in the DA neurons [42, 63]. Accordingly, a larger fraction of VTA-residing DA neurons will acquire tonic activity. Moreover, the firing frequency of a fraction of tonically-firing DA neurons may also rise as well as the fraction of burst DA firing neurons may also increase during delay period. These different processes would together be responsible for the stable (tonic) increase in population-averaged activity of mesocortical DA neuron sub-population in the VTA in response to increase in sustained-firing activity in the PFC. Therefore, it is strongly possible that the tonic activity of the DA sub-population strictly involved in the closed-loop mesocortical circuit may increase due to the sustained-firing activity in the PFC. Consequently, it may lead to enhanced tonic DA release in the cortex and underlies WM maintenance during delay period.

Accordingly, the variations in the population-averaged activity of mesocortical DA neurons, *a*_*DN*_, in the VTA and the cortical bulk or volume-averaged extracellular DA concentration or content, [*DA*], under the mesoencephalic excitation are modeled here as,

$$\begin{array}{c} \frac{da_{DN}(t)}{dt}=-\frac{\Delta a_{DN}(t)}{\tau_{DN}}+W_{PD}f(c_{1}\Delta a_{PN}) \end{array}$$ (4)

$$\begin{array}{c} \frac{d\left[DA\right](t)}{dt}=-\frac{\Delta \left[DA\right](t)}{\tau_{DA}}+R_{DA}f(c_{3}\Delta a_{DN}) \end{array}$$ (5)

Where, $\Delta a_{DN}(t)=a_{DN}(t)-a_{DN}^{basal}$ and Δ[*DA*](*t*) = [*DA*](*t*) − [*DA*]^*basal*^. The $a_{DN}^{basal}$ and [*DA*]^*basal*^ corresponds to the basal activity of mesocortical DA neurons and the basal extracellular DA concentration, respectively, in the PFC under resting conditions.

The first term on the right-hand side of Eq 4 denotes the excitability of the population of DA neurons characterized by the specific time constant *τ*_*DN*_. A large time constant implies a greater excitability of the DA neurons. The second term in Eq 4 represents the excitation of DA neurons by the cortical pyramidal activity *a*_*PN*_ with the glutamatergic synaptic efficacy *W*_*PD*_. Further, the first term in Eq 5 represents the uptake and degradation of DA in the extracellular region in the PFC with the characteristic time constant *τ*_*DA*_ whereas the second term signifies the release of DA by the excited DA neuron population with the efficiency parameter, *R*_*DA*_. *R*_*DA*_ denotes the DA-releasability of the dopaminergic projections and critically relies on the intrinsic DA metabolism and release probability of the DA-containing vesicles at the axonal terminals of mesocortical projections (Fig 1B).

Anatomical and electrophysiological studies have shown that there also exists a population of GABAergic neurons in the VTA which receives glutamatergic inputs from the cortical areas and acts as a brake system to suppress the excess activity of the DA neuron population [64]. The present model does not incorporate an explicit dynamics of GABA population in the VTA. Rather, the magnitudes of the parameters *W*_*PD*_ for excitation of DA neurons by cortical projections and *τ*_*DN*_ for the self-decay of DA population activity have been adjusted in a manner so that the putative effects of VTA-inhabiting GABA population could be accounted for. Somatodendritic D2 autoreceptors are generally known to play a crucial role in lateral inhibition of DA neuron activity in the VTA. However, the sub-population of DA neurons in the VTA extending mesocortical projections stands as an exception to this phenomenon of somatodendritic lateral inhibition [65]. Furthermore, the cortical DA content has been assumed here as a single entity or a pool which varies according to DA neuron’s activity. An explicit consideration of synaptic release of DA and its volume diffusion in the cortical area is ignored to satisfy the neural mass framework of the model.

### Modeling the effect of D1R activation on cortical excitability and synaptic transmission

In the presence of extracellular DA in the PFC, D1R activation causes modulation of the activity of several voltage-gated and ligand-gated ionotropic receptors [66] located on the cortical neurons. Consequently, this leads to the modulation of neuronal excitability of the pyramidal neurons [67] and GABAergic interneurons [68] as well as the modulation of the excitatory [69] and inhibitory [70] synaptic efficacies in the local cortical network (Fig 1C). However, the resultant level of cortical D1R stimulation in response to cortical DA level further depends on the parameter, D1R-sensitivity [26]. It signifies how efficiently the cortical network perceives any change in DA content and, hence, depends collectively on the cortical D1R density and the reactivity of DA-binding sites on individual D1Rs (Fig 1B). Therefore, the resultant level of D1R activation or stimulation, *D*1*R*_*act*_, in the presence of cortical DA content [*DA*] is modeled here as,

$$\begin{array}{c} D1R_{act}(t)=D1R_{sens}f(c_{4}\Delta \left[DA\right]) \end{array}$$ (6)

where, *D*1*R*_*sens*_ signifies the D1R-sensitivity of the cortical neurons to cortical DA pool. Further, the dopaminergic modulation of the neuronal excitability and the synaptic efficacies in the cortical neuronal populations in response to D1R stimulation is given by,

$$\begin{array}{c} \tau_{IN}=\tau_{IN}^{*}(0.24D1R_{act}+0.26) \end{array}$$ (7)

$$\begin{array}{c} W_{PP}=W_{PP}^{*}(0.12D1R_{act}+0.68) \end{array}$$ (8)

$$\begin{array}{c} W_{PI}=W_{PI}^{*}(0.12D1R_{act}+0.68) \end{array}$$ (9)

where, $\tau_{IN}^{*}$, $W_{PP}^{*}$ and $W_{PI}^{*}$ are the basal magnitudes of the respective parameters. Notably, the strengths of the parameters *W*_*PP*_, *W*_*PI*_ and *τ*_*IN*_ are modeled here to linearly increase with the increase in *D*1*R*_*act*_.

D1R stimulation leads to increase in the excitability of GABAergic interneurons by causing decrease in the potassium channel conductance [68]. Therefore, increase in *τ*_*IN*_ with the increase in *D*1*R*_*act*_ leads to slower spontaneous decay of the activity of interneuron population and reflects increase in the population excitability. Further, at excitatory synapses, D1R stimulation causes increase in the conductance and decay time constant of the NMDA receptors whereas it leads to slight reduction in the AMPA receptor-mediated postsynaptic currents [69]. In fact, this effect on NMDA receptors is pivotal to the robust sustained-firing activity in the cortical network [71, 72]. As mentioned above, *W*_*PP*_ and *W*_*PI*_, both are the strengths of excitatory synapses involved in the recurrent excitation of pyramidal neurons and the excitation of inhibitory interneurons, respectively. It is evident that these synaptic efficacies as such do not differentiate between the AMPA and NMDA receptor-mediated synaptic currents. However, the increase in *W*_*PP*_ and *W*_*PI*_ with rising *D*1*R*_*act*_ is meant to achieve the increase in excitatory synaptic transmission naturally occurring due to the prolonged charge transfer under the increased NMDA receptor conductance as well as time constants of the NMDA receptor-mediated currents. This efficiently leads to the enhancement of the self-excitation of pyramidal population and the excitation of interneuron population, which engenders sustained-firing activity in the present modeling framework. Accordingly, the increase in the synaptic efficacies with the increase in D1R stimulation manifests into the form of synaptic plasticity [13]. Nonetheless, D1R stimulation also causes increase in the excitability of pyramidal neurons by decreasing the threshold of depolarization by the persistent sodium current (*I*_*NaP*_) and simultaneously reduces the inactivating potassium currents (*I*_*K*^+^_) [13]. However, contrary to the case of interneuron excitability, the parameter *τ*_*PN*_, representing pyramidal population excitability, has not been conceived here to increase with increase in *D*1*R*_*act*_. Rather, this effect is compensated through an appropriate magnification of *W*_*PP*_. Owing to the fact that the pyramidal population has a term of self-amplification of their activity, decrease in spontaneous decay of its population activity under high excitability can be conceived through relatively stronger recurrent excitation and an additional term of *D*1*R*_*act*_-dependence could be dropped for the tractability of the model.

Therefore, the present model not only considers the direct modulation of pyramidal neurons through D1Rs located on them but also indirect modulation through GABAergic transmission. A glossary of the key variables and the free parameters of the model is available in Table 1.

**Table 1:** The definitions of the key dynamical variables and the free parameters of the closed-loop mesocortical model.

Variables\Parameters	Definitions
**a_PN_**	Average activity (in *Hz*) of the population of excitatory pyramidal neurons in the local cortical network in DLPFC during delay period. **(Variable)**
**a_IN_**	Average activity (in *Hz*) of the population of inhibitory GABAergic interneurons in the local cortical network in DLPFC during delay period. **(Variable)**
**a_DN_**	Average activity (in *Hz*) of the population of DA neurons in the VTA extending mesocortical projections to the DLPFC. It maintains tonic release of DA during delay period. **(Variable)**
[**DA**]	Delay-associated bulk extracellular DA concentration (in *nM*) of the DLPFC. **(Variable)**
**R_DA_**	DA-releasability (in *nM*.*ms*^−1^) from the mesocortical afferents in the DLPFC. It signifies the efficiency of tonic release of DA from the dopaminergic projections during delay period and depends on the DA metabolism as well as release probability of DA-containing vesicles at the axonal terminals. **(Parameter)**
**D1R_act_**	Resultant level (in *A*.*U*.) of D1R activation or stimulation in the local cortical network during delay period. **(Variable)**
**D1R_sens_**	D1R-sensitivity (in *A*.*U*.) of the local cortical network in the DLPFC. It signifies the efficiency of the cortical neurons to sense variation in cortical DA content and depends on the D1R density as well as reactivity of DA-binding sites. **(Parameter)**

The magnitudes of the various parameters in the model are available in Table 2. The parameters of the cortical dynamics have been computed by establishing equivalence of the system of coupled differential equations (Eqs 1 and 2) for cortical neuronal populations to the set of differential equations for population-activities described in the mean-field approach by Brunel and Wang [7]. The remaining parameters of the dynamics of DA neuron population, DA release and D1R stimulation are calibrated in a trial-based manner to acquire the modulation output of the cortical activities known during delay [7] and of the associated empirical observations of cortical DA level [49].

**Table 2:** List of parameters present in the mathematical model and its stochastic framework along with their values. The parameters with values in bold font are the free parameters varied in the present study.

Parameters	Values	Units
$a_{PN}^{basal}$	3	*Hz*
$a_{IN}^{basal}$	9	*Hz*
$a_{DN}^{basal}$	3	*Hz*
[*DA*]^*basal*^	0.2	*nM*
$W_{PP}^{*}$	8.5077	*Hz*.*ms*^−1^
$W_{PI}^{*}$	6.4570	*Hz*.*ms*^−1^
*W*_*PD*_	3.2790	*Hz*.*ms*^−1^
*W*_*IP*_	5.1613	*Hz*.*ms*^−1^
*W*_*II*_	0.0	*Hz*.*ms*^−1^
*R*_*DA*_	**0-0.05**	*nM*.*ms*^−1^
*D*1*R*_*sens*_	**2-10**	*A*.*U*.
*τ*_*PN*_	20	*ms*
$\tau_{IN}^{*}$	6.8	*ms*
*τ*_*DN*_	10	*ms*
*τ*_*DA*_	800	*ms*
*c*_1_	0.009852	-
*c*_2_	0.018259	-
*c*_3_	0.001052	-
*c*_4_	9.375000	-
*σ*_1_	0.76125	-
*σ*_2_	0.08215	-
*σ*_3_	0.14256	-
*σ*_4_	0.00080	-

### Equilibrium analysis and WM-robustness

**Fig 2:**
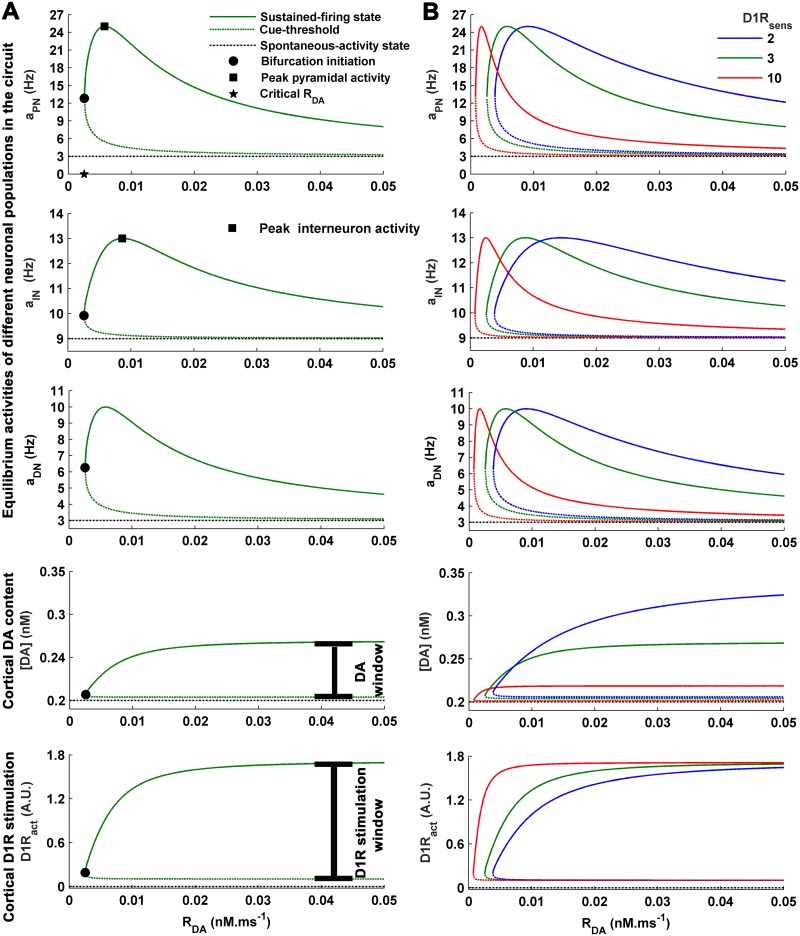
The delay-associated state of the mesocortical dynamics is characterized by the global equilibrium state of its various dynamical elements. (A) Given a fixed value of D1R-sensitivity *D*1*R*_*sens*_ (here *D*1*R*_*sens*_ = 3, normal control), the bifurcation profiles of the dynamical elements are shown with DA releasability *R*_*DA*_ as the bifurcation parameter. Critical *R*_*DA*_, and the corresponding critical cortical dopamine content [*DA*] and D1R stimulation level *D*1*R*_*act*_, mark the beginning of bistable regime favoring the working memory maintenance during delay period. The higher stable states of the bifurcation profiles are together associated with the sustained-firing state of the cortical dynamics whereas the lower stable states together signify the basal spontaneous-activity state. The ranges of [*DA*] and *D*1*R*_*act*_ spanned by their higher stable states represent the spans or windows of cortical DA content and D1R stimulation, respectively, underlying the entire modulation profile of the cortical dynamics. The maximum limit to which [*DA*] or *D*1*R*_*act*_ may may increase with increase in *R*_*DA*_ marks the saturation level. The cue-threshold in the *a*_*PN*_ bifurcation profile signifies the minimum excitation of the pyramidal population by cue input, which causes switching to the sustained-firing state. (B) Alteration in *D*1*R*_*sens*_ further affects the bifurcation profiles. Most prominently, increase in *D*1*R*_*sens*_ causes leftward shift of the bifurcation region.

The delay-associated state of the mesocortical dynamics is characterized by its global steady or equilibrium state, which is defined as

$$\begin{array}{c} \frac{d\vec{x}(t)}{dt}=0 \end{array}$$ (10)

where $\vec{x}(t)=[a_{PN}(t),a_{IN}(t),a_{DN}(t),\left[DA\right](t)]$ and represents the set of state-variables. In this regard, the nullcline plots of the state-variables *a*_*PN*_ and *D*1*R*_*act*_ in the *a*_*PN*_-*D*1*R*_*act*_ state-space are obtained first (S1 Fig). The intersection points of the *a*_*PN*_- and *D*1*R*_*act*_-nullclines define the operational points of mesocortical dynamics during the delay period for a given set of parameters *R*_*DA*_ and *D*1*R*_*sens*_. Accordingly, the nullcline analysis facilitates the obtainment of the bifurcation plots of the state-variables by varying *R*_*DA*_ under a fixed *D*1*R*_*sens*_ (Fig 2).

Further, the mesocortical dynamics is constantly affected by the various natural sources of noise in the neural system [73]. Therefore, the stochastic framework of the mesocortical dynamics [36] is given by,

$$\begin{array}{c} da_{PN}=f_{PN}(\vec{x})+\sigma_{1}dW_{1} \end{array}$$ (11)

$$\begin{array}{c} da_{IN}=f_{IN}(\vec{x})+\sigma_{2}dW_{2} \end{array}$$ (12)

$$\begin{array}{c} da_{DN}=f_{DN}(\vec{x})+\sigma_{3}dW_{3} \end{array}$$ (13)

$$\begin{array}{c} d\left[DA\right]=f_{DA}(\vec{x})+\sigma_{4}dW_{4} \end{array}$$ (14)

Here, $f_{PN}(\vec{x}),f_{IN}(\vec{x}),f_{DN}(\vec{x}),f_{DA}(\vec{x})$ represent the right-hand sides of the Eqs (1), (2), (4) and (5), respectively. {*dW_i_* (*t*), *t* ≥ 0}, (*i* = 1, 2, 3, 4), denotes the Wiener process increment to each state-variable during their noisy temporal-evolution and *σ*_*i*_, (*i* = 1, 2, 3, 4), represents the corresponding noise-intensity. The magnitudes of the noise-intensities applied here are available in the Table 2 and are kept conserved throughout the study. The noise causes the state of the system to diffuse around its deterministic response and the state-variables are essentially characterized by their statistical distributions in the state-space.

To gain insight into the WM-robustness during delay period, a global potential landscape of the stochastic mesocortical dynamics is constructed. For this, the steady-state marginal probability distributions of the state-variables *P*_*st*_(*x*), where *x* ∈ {*a*_*PN*_, *a*_*IN*_, *a*_*DN*_, [*DA*], *D*1*R*_*act*_}, are obtained from the numerical simulation of the stochastic mesocortical dynamics using the Euler-Maruyama scheme [74] with a fixed time-step Δ*t*. Consequently, a joint probability distribution *P*_*st*_(*a*_*PN*_, *D*1*R*_*act*_) is obtained over the state-space *a*_*PN*_-*D*1*R*_*act*_ and the global potential landscape, *U*(*a*_*PN*_, *D*1*R*_*act*_) of the stochastic mesocortical dynamics is constructed as [36],

$$\begin{array}{c} U(a_{PN},D1R_{act})∼-\ln(P_{st}(a_{PN},D1R_{act})) \end{array}$$ (15)

The landscape is comprised of two basins of attractions associated with the spontaneous-activity state and the sustained-firing state of the mesocortical dynamics. The robustness of the WM-associated circuit dynamics is analyzed based on the two physical measures, potential barrier (PB) and the signal-to-noise ratio (SNR) of the pyramidal activity *a*_*PN*_, related with the geometry of the basin associated with the sustained-firing state. PB signifies the depth of the basin from the crest potential separating the two basins of attraction in the landscape and can be directly obtained from the *U*(*a*_*PN*_, *D*1*R*_*act*_). However, the SNR is affected by the girth of the basins and is given by,

$$\begin{array}{c} SNR=\frac{\mu }{\sigma } \end{array}$$ (16)

where, *μ* denotes the mean of the *a*_*PN*_ distribution and corresponds to the deterministic equilibrium magnitude of *a*_*PN*_ associated with the sustained-firing state and *σ* denotes the standard deviation of the noisy fluctuations around the mean *a*_*PN*_. The mathematical analysis and numerical simulations have been performed in MATLAB (The MathWorks). The scripts for the nullcline analysis, the bifurcation profiles and the numerical simulation of the stochastic dynamics are available on the ModelDB, https://senselab.med.yale.edu/modeldb/ShowModel.cshtml?model=240382.

## Results

### Features of mesocortical dynamics facilitating WM maintenance during delay

The parameters DA-releasability (*R*_*DA*_) and D1R-sensitivity (*D*1*R*_*sens*_) of the model serve as the free parameters or handles for realizing here the alterations in cortical DA content and sensitivity, respectively. Notably, *R*_*DA*_ signifies the volume-averaged rate or efficiency of DA influx from dopaminergic projections into the cortical extracellular space. Although change in DA-releasability has indeed been observed to affect cognitive performance in the earlier studies involving administration of psychostimulant drugs such as amphetamine and phencyclidine [11, 75], the exact quantification of this rate of DA influx could not have been possible. Accordingly, *R*_*DA*_ is varied here within a range of 0.00–0.05*nM*.*ms*^−1^, which is found suitable to capture the experimentally-observed profile of DA-dependent modulation of cortical persistent activity [7] within the present model framework.

Similarly, *D*1*R*_*sens*_ regulates the sensing-end of the process of dopaminergic transmission. Although D1R-sensitivity is experimentally measured in terms of BP (a dimensionless quantity), alteration in *D*1*R*_*sens*_ has been scaled here to an integer interval of 2–10. It must be noted that alteration in D1R-sensitivity does not generally imply alterations in the intracellular signalling of D1R activation. Therefore, the parameters in Eqs 7–9, which govern the excitability of neuronal populations and excitatory or inhibitory synaptic efficacies in response to a given D1R stimulation level (*D*1*R*_*act*_), remain unaffected when *D*1*R*_*sens*_ is varied.

The study begins here with noting the salient features of the delay-associated responses of the various quantifiable variables embedded in the proposed dynamical framework towards change in *R*_*DA*_, while the other free parameter *D*1*R*_*sens*_ is kept fixed at a particular value. This configuration would physiologically correspond to the change in cortical DA content and the associated changes in the cortical as well as VTA neuron dynamics, under a control normal D1R-sensitivity of the cortex within the present modeling framework. Fig 2A shows the firing frequencies of different neuronal populations (*a*_*PN*_ for pyramidal neurons, *a*_*IN*_ for interneurons and *a*_*DN*_ for DA neurons), extracellular cortical DA level ([*DA*]) and level of cortical D1R stimulation (*D*1*R*_*act*_) during delay period at different values of *R*_*DA*_, for the *D*1*R*_*sens*_ = 3. The profile of each quantity exhibits a bifurcation behavior. The set of lower values provide the basal magnitude of the quantity associated with spontaneous-activity state in the cortex whereas that of the higher values provide the magnitude associated with sustained-firing activity. The monostable region is characterized by a single stable equilibrium state associated with spontaneous-activity in the cortex. Hence, for the values of *R*_*DA*_ within the monostable region, sustained-firing in the cortex is biophysically not feasible. Only in the bistable region, sufficiently strong cue stimulus can cause the switching of the mesocortical dynamics to the sustained-firing state. Therefore, the initiation point of bifurcation signifies the critical *R*_*DA*_, which marks the boundary of phase transition from a region devoid of sustained firing to that of WM maintenance. Accordingly, [*DA*] and *D*1*R*_*act*_ associated with the critical *R*_*DA*_ indicate the critical DA content and D1R stimulation level required to commence the regime of sustained firing. Notably, *R*_*DA*_ naturally comes forth as the bifurcation parameter because its variation, under a fixed *D*1*R*_*sens*_, leads to change in [*DA*] and associated *D*1*R*_*act*_, which eventually causes modulation of the neuronal activities during delay.

Remarkably, the increase in *R*_*DA*_ does not lead to an unlimited increase in the sustained firing-associated [*DA*] and *D*1*R*_*act*_ during delay. The maximum level to which they may rise is marked by their unique saturation levels (Fig 2A). This limitation is of purely functional nature imposed by the mesocortical dynamics during steady-state of the sustained-firing activity in cortex. Therefore, together with the critical [*DA*] and *D*1*R*_*act*_, the corresponding saturation levels define the spans or windows of cortical DA content and D1R stimulation, respectively, which underlie the entire dopaminergic modulation profiles of the neuronal activities in the bistable region.

Nonetheless, in the bistable region, the modulation profile of sustained *a*_*DN*_ activity remains in phase with that of the *a*_*PN*_ (Fig 2B) as it is the pyramidal activity which directly governs the excitation of DA neuron subpopulation in the VTA within the present mesocortical framework (Fig 1C). However, there exists a phase-lag between the modulation profiles of sustained *a*_*PN*_ and *a*_*IN*_ activities. In fact, this has also been noted in the earlier studies [33, 76] and the increase in the interneuron excitability by D1R stimulation has been proposed to lag behind that of the pyramidal neurons with respect to increase in cortical DA content and D1R stimulation level.

The levels of spontaneous and sustained activities of the various types of neuronal populations involved here closely resemble their empirically-known estimates during delay. *a*_*PN*_ and *a*_*IN*_ display spontaneous activities at 3*Hz* and 9*Hz*, respectively, during delay (Fig 2A), which are of the order of the average spontaneous activities of pyramidal neurons and fast-spiking GABAergic interneurons observed in the experiments carried out by Wilson et al. [77] on monkeys performing oculomotor tasks. Similarly, the modulation profiles of the sustained-firing activities (the higher stable states) in these neuronal populations span the frequency ranges 13–25*Hz* and 10–13*Hz*, respectively, which are in concordance with the earlier computational studies by Compte et al. [78] and Brunel and Wang [7] involving detailed neural network simulations. Moreover, the experimental study by Tsujimoto and Sawaguchi [79] involving delayed WM tasks also provides a similar range of these modulation profiles.

DA neurons in the VTA have been experimentally recorded to fire tonically at an approximate frequency of 3–4*Hz* under basal or resting condition in delayed-response tasks [40, 42, 63]. Accordingly, the firing rate of the spontaneous activity in DA population *a*_*DN*_ is obtained here at 3*Hz*. As argued above, the tonic firing activity in the VTA-residing DA neuron subpopulation closely associated with a local cortical network may increase in response to the sustained activity in the DLPFC during delay. However, it is also demanded that this increase should remain under the bound of the maximum tonic frequency of 10*Hz* noted earlier [63]. Therefore, the modulation profile of the sustained tonic *a*_*DN*_ is observed here to span a frequency range limited by 10*Hz* (Fig 2A).

The basal DA concentration in the spontaneous-activity state is obtained here as [*DA*] = 0.2*nM* (Fig 2A), which is close to the basal DA concentrations observed in the micro-dialysis studies performed by Watanabe et al. [49] (0.098 ± 0.013*nM*) and Jedema et al. [75] (0.31 ± 0.03*nM*) on primates during resting conditions. [*DA*] associated with sustained-firing activity in the cortex during delay (higher stable state) is observed to increase with rise in *R*_*DA*_ (Fig 2A). In this regard, Watanabe et al. [49] reported approximately 17% increase in the DA concentration in the DLPFC of healthy monkeys performing more than 98% successful trials during delayed alternation tasks. This increase in DA characterizes an optimum WM maintenance, which is also found to be associated with optimum strength or frequency of sustained-firing activity in the cortex during delay interval [80]. Accordingly, the peak *a*_*PN*_ sustained-activity coincides here with [*DA*] = 0.234*nM* (Fig 2A), equivalent to the DA increase reported by Watanabe et al. [49] under optimum performance, only for *D*1*R*_*sens*_ = 3. Therefore, the corresponding *R*_*DA*_ = 0.0058*nM*.*ms*^−1^ and *D*1*R*_*sens*_ = 3 together portray a normal healthy control in terms of the free parameters of the present model framework. Any increase or decrease in these values of *R*_*DA*_ and *D*1*R*_*sens*_ would represent an altered condition of DA-releasability and D1R-sensitivity, respectively. Subsequently, the effects of alteration in the cortical D1R-sensitivity on WM maintenance are observed through the effects on the abovementioned features of the mesocortical dynamics.

### Effects of variation in D1R-sensitivity on cortical DA level and modulation of neuronal activities

**Fig 3:**
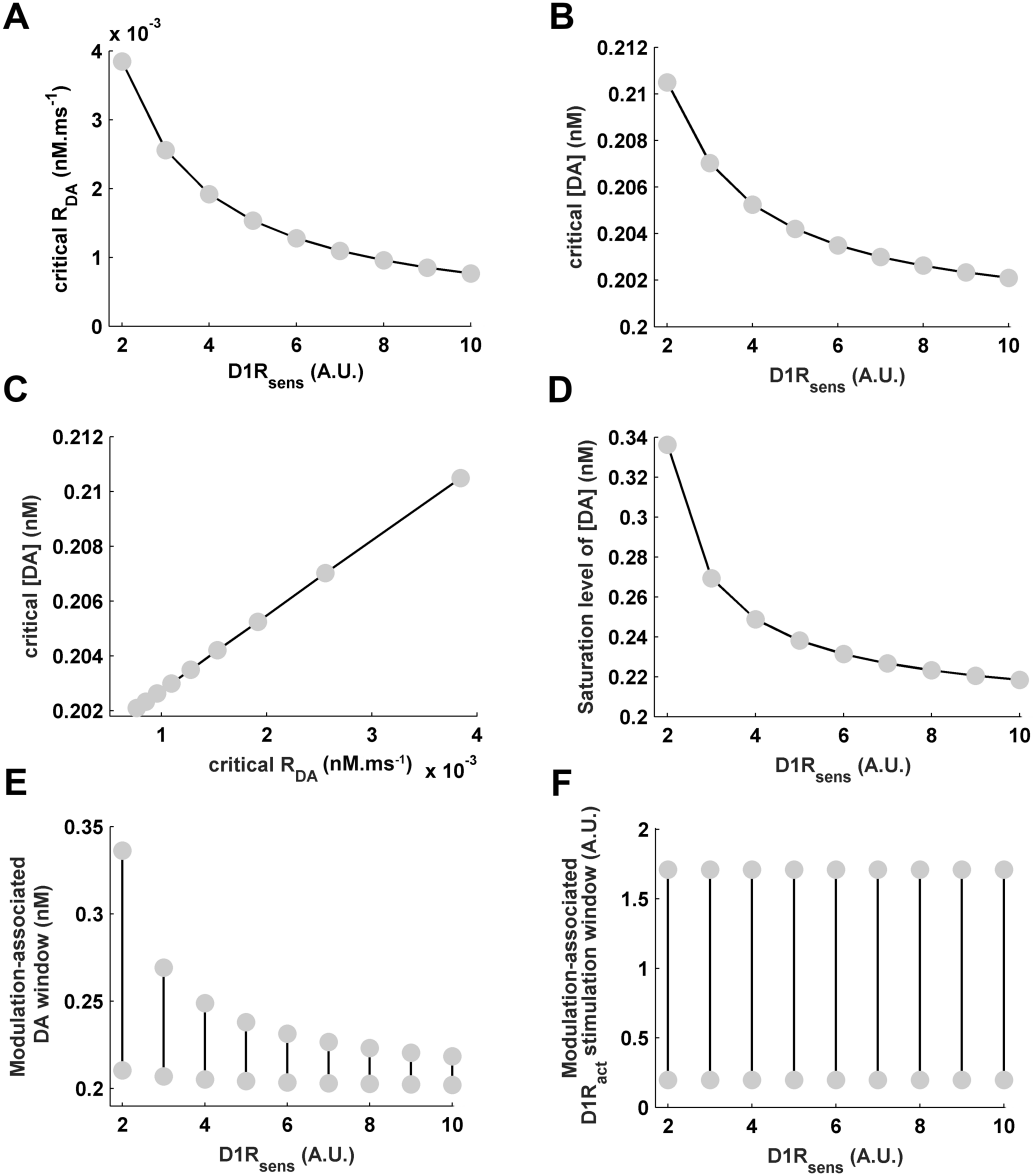
Effects of variation in D1R-sensitivity on the critical DA releasability, on the critical as well as saturations levels of cortical DA content, and on the modulation-associated windows of DA content and D1R stimulation. Increase in *D*1*R*_*sens*_ causes significant decrease in the critical *R*_*DA*_ (A) and [*DA*] (B) marking an early beginning of the bifurcation regime. The variations in critical *R*_*DA*_ and [*DA*] (C) exhibit a strong positive correlation. Moreover, the saturation level of [*DA*] (D) significantly decreases with increase in *D*1*R*_*sens*_, causing the modulation-associated window of DA (E) to shift to lower values as well as shrinks in its span. However, the modulation-associated window of D1R stimulation (F) does not vary with change in *D*1*R*_*sens*_.

Variation in *D*1*R*_*sens*_ significantly affects the bifurcation plots (Fig 2B). Its increase causes leftward shift of the profiles to lower DA-releasability (*R*_*DA*_). Consequently, increase in *D*1*R*_*sens*_ leads to a considerable decrease in the critical *R*_*DA*_ and the critical [*DA*] (Fig 3A & 3B). Notably, the variations in critical *R*_*DA*_ and [*DA*] follow a strong positive correlation (Fig 3C) depicting a tight causality-relationship between them. For the control *D*1*R*_*sens*_ = 3, the critical [*DA*] = 0.207*nM*. However, the critical [*DA*] decreases by 30% when *D*1*R*_*sens*_ is increased to 10 whereas increases by 50% across unit reduction in the control *D*1*R*_*sens*_. This leftward shift of the bifurcation profiles is due to the enhanced sensitivity of D1Rs to respond even to a less amount of DA in the surrounding medium, a consequence also hypothesized earlier for increased D1R density [23, 24, 26], and signifies possibility of WM-associated sustained activity even at lower cortical DA levels. Interestingly, the amount of leftward shift observed by increasing *D*1*R*_*sens*_ from the normal control level of 3 to 10 is equivalent to that of the rightward shift occurring through only a unit decrease in *D*1*R*_*sens*_ from the control level. It suggests that even a slight decrease in the cortical D1R-sensitivity may mark a stronger impact on the WM maintenance than a relatively significant increase in the sensitivity.

Further, increase in *D*1*R*_*sens*_ significantly reduces the [*DA*] saturation level (Figs 2B and 3D). As a result, due to the concomitant decrease in the critical as well as saturation levels of [*DA*], the DA window underlying the entire modulation phenomenon in the bistable region shifts to lower values and also shrinks in its span (Fig 3E) with rise in D1R-sensitivity. With respect to the control *D*1*R*_*sens*_ = 3, there occurs almost 27% decrease in the size of modulation-associated [*DA*] window when *D*1*R*_*sens*_ is increased to 10. At the same time, the amount of shift of the window to lower [*DA*] is almost 30%, which is the percentage decrease in the critical [*DA*] mentioned above. However, the window size increases by almost 200% (i.e. doubles in size) when *D*1*R*_*sens*_ is reduced to 2.

These observations clearly describe the impact of cortical D1R density on the regulation of DA release under the local administration of pyschostimulants in the cortical region studied by Tanaka and Okada [81]. Their study shows that, when the cortical D1R density is upregulated, the DA release is significantly reduced owing to the declined pyramidal activity. As a result, this does not allow the psychostimulants to cause any increase in the cortical DA content. Therefore, the cortical region intrinsically tends to attain a hypodopaminergic situation, which is illustrated here as the shift of modulation profiles to lower *R*_*DA*_ and the shift of modulation-associated DA window to lower DA levels.

The critical and the saturation levels of *D*1*R*_*act*_ remain unaffected (Fig 2B) from changing *D*1*R*_*sens*_. Therefore, the D1R stimulation window underlying the entire modulation phenomenon in the bistable region remains completely unaffected (Fig 3F) from *D*1*R*_*sens*_ alterations. Instead, it only influences how sharply the *D*1*R*_*act*_ responds to the change in [*DA*] associated with variation in *R*_*DA*_ and reaches its saturation level (Fig 2B).

**Fig 4:**
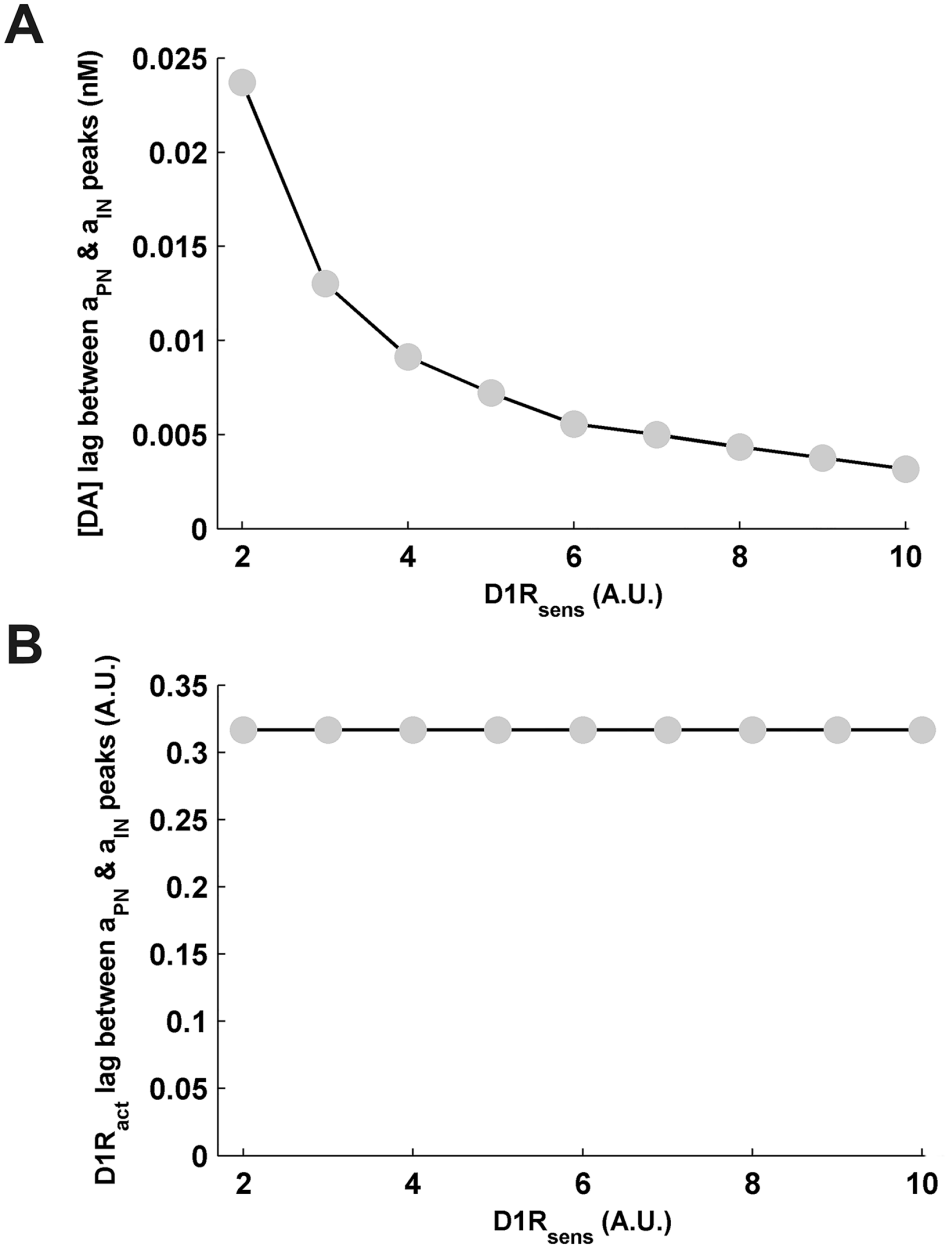
Effects of variation in D1R-sensitivity on the phase-lag between the dopaminergic modulation profiles of sustained pyramidal and interneuron activities. (A) The phase-lag between the peak *a*_*PN*_ and the peak *a*_*IN*_ activities with respect to the associated [*DA*] levels is seen to considerably decrease with increase in *D*1*R*_*sens*_ signifying a steeper modulation of the neuronal activities with unit change in [*DA*]. (B) However, the phase-lag with respect to the associated *D*1*R*_*act*_ levels does not vary.

The observed effects of D1R-sensitivity on the modulation-associated DA and D1R windows further noticeably influences the modulation profiles of delay-associated sustained activities in the different neuronal populations. Owing to the invariant *D*1*R*_*act*_, the respective ranges of magnitude spanned by the modulation profiles of sustained activities *a*_*PN*_, *a*_*IN*_ and *a*_*DN*_, viz. 13–25*Hz*, 10–13*Hz* and 6–10*Hz*, respectively, remain conserved with the variation in *D*1*R*_*sens*_ (Fig 2B). In fact, D1R stimulation level is the immediate driver of the modulation of these neuronal activities. However, besides the leftward shift of the profiles towards lesser *R*_*DA*_ and [*DA*] noted above, the sharpness of the modulation profiles of sustained activities in response to change in *R*_*DA*_ considerably increase at higher *D*1*R*_*sens*_ across all neuronal populations. Moreover, the phase-lag between the peak sustained *a*_*PN*_ and *a*_*IN*_ activities in terms of [*DA*] significantly decreases with increase in *D*1*R*_*sens*_ (Fig 4A) and indicates lesser difference in the cortical DA required for D1R-mediated enhancement of the pyramidal and interneuron excitability. This decrease in phase-lag essentially emanates from the observed shrinkage in the DA span underlying modulation (Fig 3E) at higher *D*1*R*_*sens*_. However, the phase-lag with respect to *D*1*R*_*act*_ remains unaffected (Fig 4B), again due to the absence of effect of *D*1*R*_*sens*_ on modulation-associated *D*1*R*_*act*_ span. This decrease in the phase-lag and increase in the sharpness of the modulation profiles of sustained-activities in the neuronal populations at higher D1R-sensitivity critically affect the optimal range of cortical DA content underlying optimal WM maintenance, as described below.

**Fig 5:**
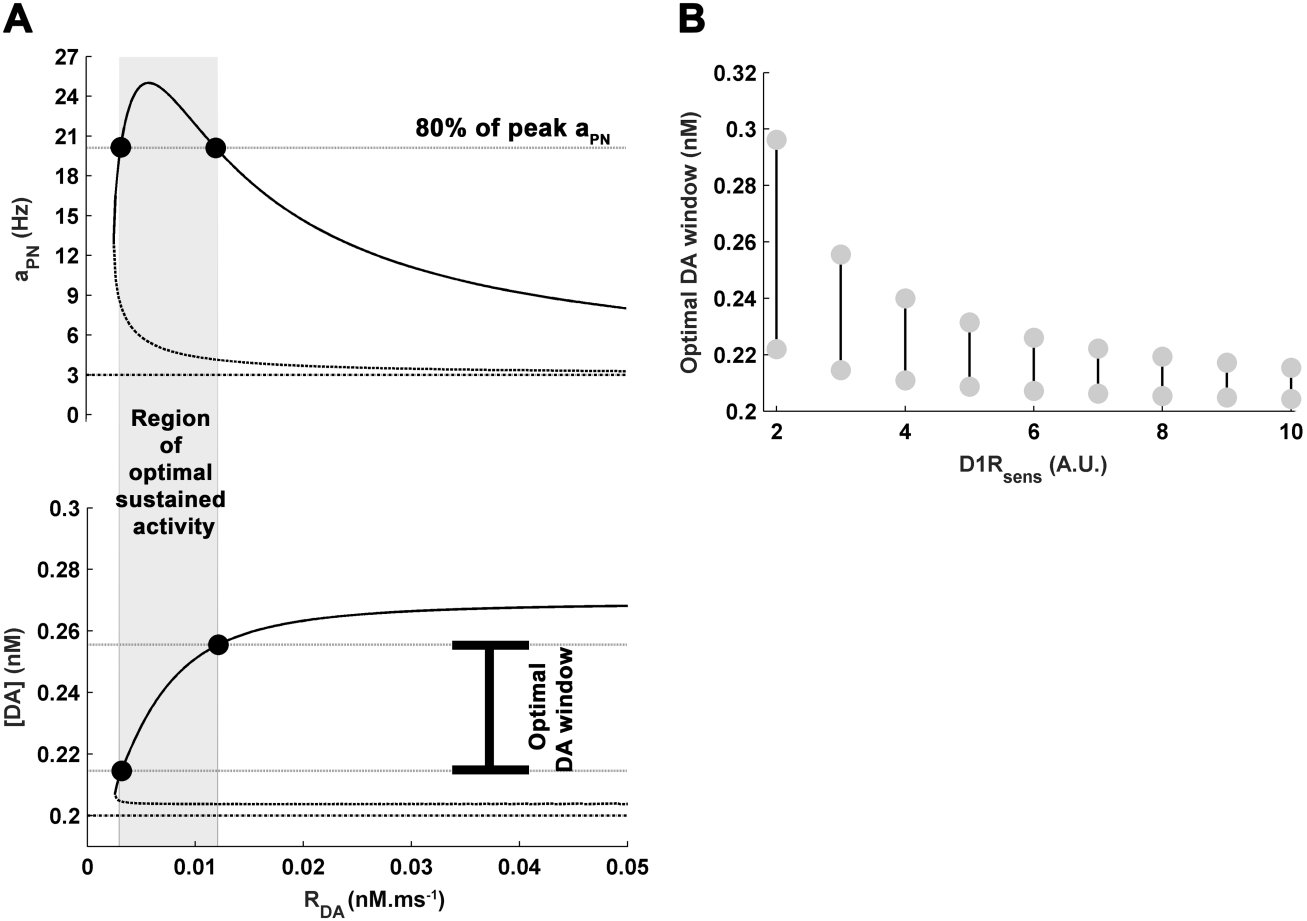
Effects of variation in D1R-sensitivity on the range of optimal DA facilitating optimal WM maintenance. (A) An illustration for the concept of optimal DA range or window associated with the region of optimal sustained *a*_*PN*_ activity. It is assumed here that the sustained pyramidal activity above 80% of the peak activity in the modulation profile facilitates efficient WM maintenance. (B) The optimal DA window is seen to considerably shrink and shift to lower values as the *D*1*R*_*sens*_ is increased.

In the earlier studies involving D1R agonists and antagonists, it has been noted that the strength of sustained-firing activity [32, 82] and WM performance [11, 12] both exhibit inverted-U shaped profile with variation in the level of D1R stimulation. Accordingly, both are highly correlated such that a poor performance is often associated with poor persistent activity in the PFC [33]. Recent studies have provided strong evidences for a linear relationship between them [80, 83, 84]. Accordingly, a symmetric span around the peak sustained *a*_*PN*_ activity in the modulation profile (Fig 5A) is chosen such that activity greater than or equal to 80% of the peak activity is assumed to facilitate sound WM maintenance during delay. Hence, the range of cortical DA facilitating this span of optimal sustained pyramidal activity signifies the ‘optimal DA window’. It is observed that the optimal DA window substantially shrinks with rise in *D*1*R*_*sens*_ (Fig 5B). In the present study, this optimal DA window shrinks to 30% of the normal control with increase in *D*1*R*_*sens*_ to 10.

Shrinking of optimal DA window demonstrates a smaller range of cortical DA content over which optimal WM maintenance could be acquired. Therefore, even weak natural fluctuations in DA-releasability and the resulting extracellular DA would be able to shift the dynamics to poor maintenance and may have dramatic effects on the cognitive ability. This observation supports the earlier hypothesis [23, 24, 26] that alteration in cortical D1R density has been suggested as a potential factor affecting the optimal region of WM maintenance in schizophrenia. Although the estimate (> = 80%) set here for the boundary of optimal sustained pyramidal activity is merely for the purpose of demonstration, the observation regarding narrowing of the optimal DA window with increase in D1R-sensitivity will remain unaffected regardless of the different estimates one may choose.

### Effects of variation in D1R-sensitivity on the robustness of WM maintenance

**Fig 6:**
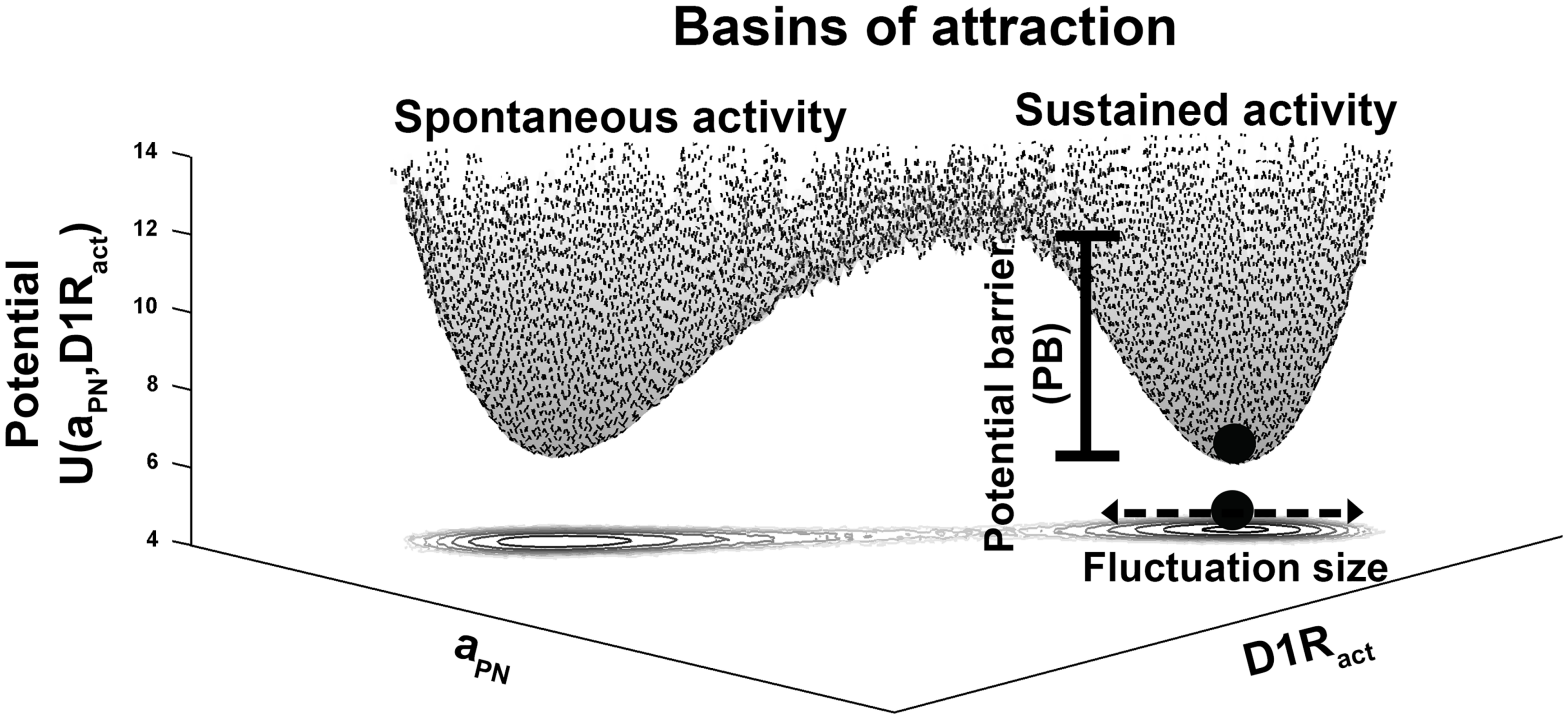
The global potential landscape of the noisy mesocortical dynamics. For the normal control parameters DA-releasability (*R*_*DA*_ = 0.0058*nM*.*ms*^−1^) and D1R-sensitivity (*D*1*R*_*sens*_ = 3) of the mesocortical dynamics, the global potential landscape is shown over the *a*_*PN*_-*D*1*R*_*act*_ plane, along with its contour projection onto the plane. The system in sustained-firing state is depicted by a ball sitting in the corresponding basin of attraction whose depth provides the potential barrier (PB) restricting the noise-induced transition of the system to spontaneous-activity state. The contour projection illustrates the fluctuation size in the system state around its mean point, which governs the signal-to-noise ratio (SNR) of the cortical sustained activity facilitating WM maintenance.

The global potential landscape (Fig 6) is procured from the steady-state of the noisy mesocortical dynamics (Eqs 11–14). The features of WM-robustness under different conditions of DA-releasability (*R*_*DA*_) and D1R-sensitivity (*D*1*R*_*sens*_) are derived simultaneously from two physical measures related with the geometry of WM-associated basin of attraction. First, the potential barrier (PB) emanates from the depth of the basin and restricts the noise-induced transition of circuit dynamics from the sustained-firing state to the spontaneous-activity state. Second, the signal-to-noise ratio (SNR) of sustained-firing activity manifests from the girth of the basin and illustrates the strength of the sustained-firing activity relative to its noise content. WM-robustness is directly proportional to both these measures.

**Fig 7:**
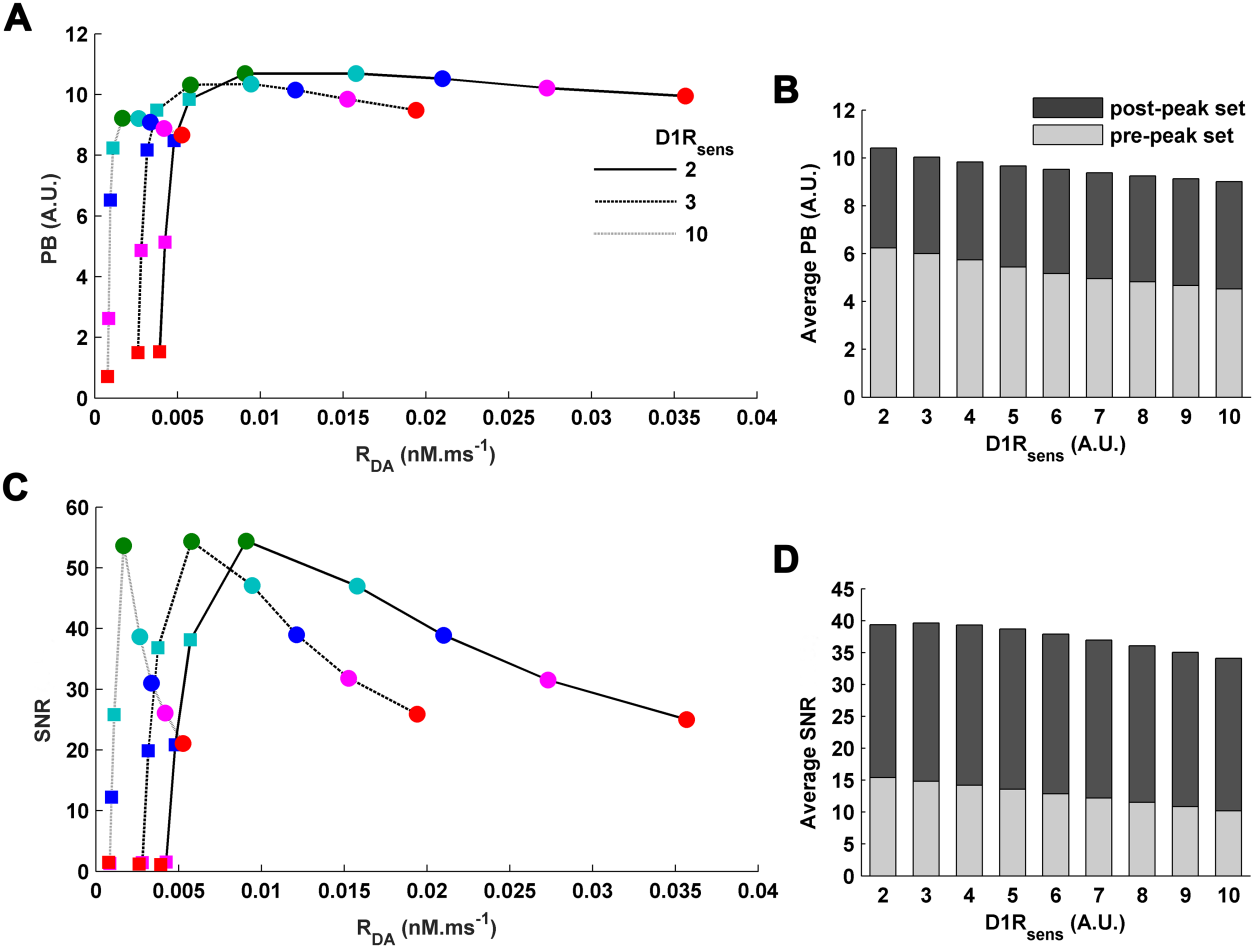
The conserved features of WM-robustness across variation in D1R-sensitivity. (A) For the different *D*1*R*_*sens*_, PB for the sampled levels of sustained pyramidal activities along the *a*_*PN*_-modulation profile always follows a concave profile. The sampled activities from the pre-peak side of the *a*_*PN*_-modulation profile are marked with color-filled squares and that from the post-peak side are shown in color-filled circles. The sampled activities, 90% (cyan), 80% (blue), 70% (magenta), 60%(red), are percentage activities with respect to the peak 100% (green) sustained activity. (B) The average PB of the post-peak set of sustained activities (including the peak activity) in the *a*_*PN*_-modulation profile is always higher than that of the pre-peak set for every *D*1*R*_*sens*_. (C) Similarly, SNR for the sampled levels of sustained pyramidal activities always follows a concave profile under different condition of *D*1*R*_*sens*_. (D) Moreover, the average SNR of the post-peak set of sustained activities is always higher than that of the pre-peak set for all values of *D*1*R*_*sens*_.

**Fig 8:**
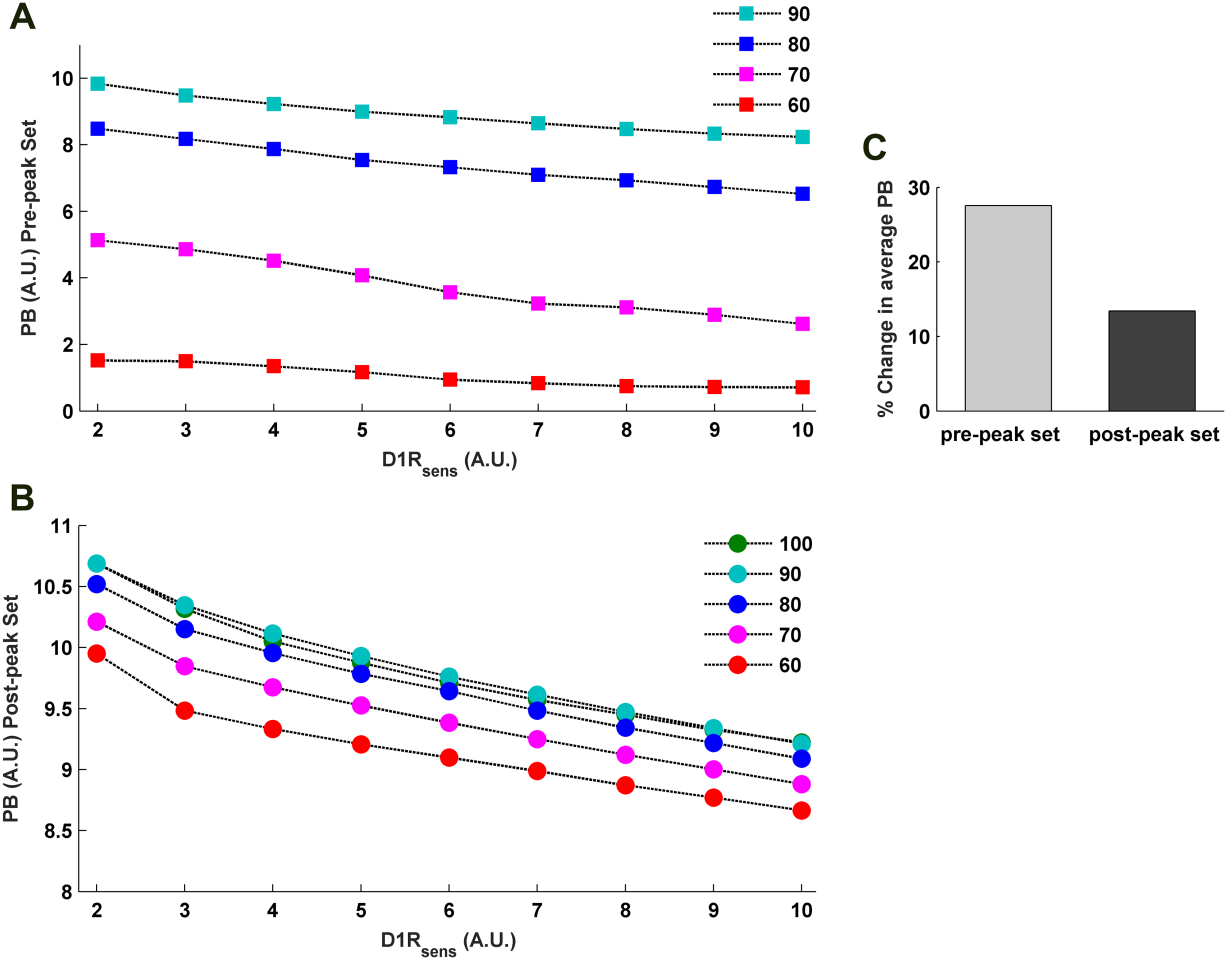
Effects of variation in D1R-sensitivity on the WM-robustness in terms of potential barrier (PB). Increase in *D*1*R*_*sens*_ causes a consistent decrease in the PB of any individual level of sustained activity either sampled from the pre-peak (A) or from the post-peak (B) set of the modulation profile of cortical sustained *a*_*PN*_ activity. The percentage activities are with respect to the peak (100%) sustained activity. (C) The percent decrease in the average PB of pre-peak and post-peak sets across increase in *D*1*R*_*sens*_ shows higher vulnerability of the pre-peak set to change in D1R-sensitivity.

**Fig 9:**
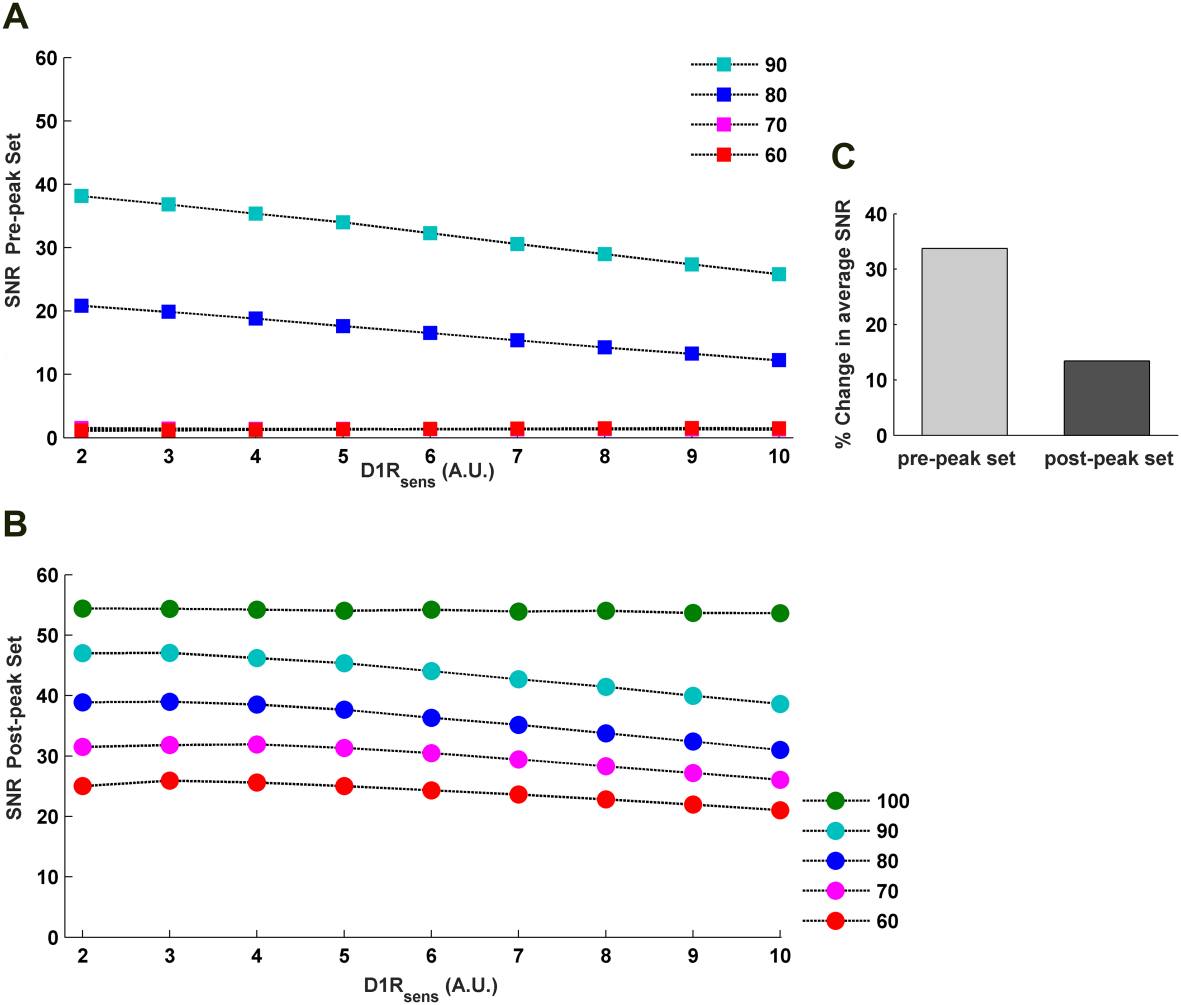
Effects of variation in D1R-sensitivity on the WM-robustness in terms of signal-to-noise ratio (SNR). Similar to the PB, increase in *D*1*R*_*sens*_ causes a consistent decrease in the SNR of any individual level of sustained activity either sampled from the pre-peak (A) or from the post-peak (B) set of the modulation profile of cortical sustained *a*_*PN*_ activity. The percentage activities are with respect to the peak (100%) sustained activity. (C) The percent decrease in the average SNR of the pre-peak and post-peak sets across increase in *D*1*R*_*sens*_ indicates higher vulnerability of the pre-peak set to change in D1R-sensitivity.

We begin with exploring the features of WM-robustness which remain intact despite alterations in *D*1*R*_*sens*_. This involves examining the effect of varying *R*_*DA*_ on the WM-robustness and essentially projects the impact of alteration in cortical DA content [*DA*] on WM-robustness mediated through change in the underlying level of D1R stimulation *D*1*R*_*act*_ during delay. Variations in PB and SNR along the modulation profile of sustained-firing activity *a*_*PN*_ in the bistable region (Fig 2A) always depict a concave profile of WM-robustness (Fig 7A & 7C), similar to the shape of the modulation profile itself. Accordingly, it illustrates a tight relationship between the firing frequency of sustained-firing activity and WM-robustness. Moreover, if the *a*_*PN*_ modulation profile is partitioned into two sections, the pre-peak set and the post-peak set (including the peak sustained activity), the average PB and SNR of the post-peak set are substantially higher than that of the pre-peak set (Fig 7B & 7D). Notably, this suggests that the post-peak set which involves higher *D*1*R*_*act*_ as well as an inhibition-dominated cortical dynamics is much more robust than the pre-peak set involving relatively lesser *D*1*R*_*act*_ and an excitation-dominated cortical dynamics (Fig 2A). These observations have a remarkable similarity with that obtained in the earlier theoretical studies [6, 7, 9, 36, 85] involving change in the D1R stimulation level assumed to occur through alteration in cortical DA content during delay. However, the present investigation further shows that these specific features also remain identically conserved across alterations in D1R-sensitivity. Furthermore, sustained *a*_*PN*_ activity more than or equal to 80% of the peak sustained activity in the *a*_*PN*_ modulation profile noticeably share high levels of WM-robustness (Figs 8A & 8B and 9A & 9B). This suggests that the optimal region in the *a*_*PN*_ modulation profile associated with the optimal DA window is not only defined by its optimal levels of sustained-firing activity but also by the optimal WM-robustness during delay.

Next, we examine the *D*1*R*_*sens*_-sensitive features of WM-robustness. The entire concave profile of robustness, either in terms of PB (Fig 7A) or SNR (Fig 7C), exhibits a downward shift to lower levels when the *D*1*R*_*sens*_ is increased. This is also seen through a consistent decrease in PB (Fig 8A & 8B) and SNR (Fig 9A & 9B) of the individual sustained-firing activities of different firing strengths sampled across the *a*_*PN*_ modulation profile. Consequently, average PB and SNR of the pre-peak as well as the post-peak set of sustained activities in the *a*_*PN*_ modulation profile also decrease. Together, these observations illustrate a concomitant rise in instability of the WM maintenance during delay with increase in *D*1*R*_*sens*_. Nonetheless, the amount of decrease in the average PB and SNR (Figs 8C and 9C) is higher for the pre-peak set in comparison to the post-peak set. This differential response to increase in *D*1*R*_*sens*_ immediately indicates that the robustness of sustained-firing activities during delay resulting from lower *D*1*R*_*act*_ and excitation-dominated excitation-dominated cortical dynamics is more vulnerable to alteration in *D*1*R*_*sens*_. However, sustained activities associated with higher *D*1*R*_*act*_ and inhibition-dominated cortical dynamics is more resistant to decrease in robustness inflicted by increase in *D*1*R*_*sens*_.

As noted above, *D*1*R*_*sens*_ does not affect the span of *D*1*R*_*act*_ which underlies the modulation of sustained neuronal activities during delay (Figs 2A and 3f). Therefore, the observed effects of *D*1*R*_*sens*_ on the WM-robustness is certainly not mediated through the known conventional mechanisms involving the D1R stimulation level [6, 7, 9]. However, what varies across the *D*1*R*_*sens*_ is the [*DA*] underlying underlying the conserved *D*1*R*_*act*_. Therefore, the observed effects on the WM-robustness appears to be essentially mediated through the impact of D1R-sensitivity on the modulation-associated DA window (Fig 3E). More specifically, it appears to arise from the shift of modulation-associated DA window to lower levels with rising sensitivity. Immediately, a completely new role of cortical DA content in shaping the WM-robustness is realized under the conditions of varying D1R-sensitivity, where sustained-firing activity acquired at a particular level of D1R stimulation but at lower cortical DA content would be lesser robust than that acquired at the same D1R stimulation but at higher cortical DA content.

## Discussion

Using the neural mass model of the prefronto-mesoprefrontal system, the present study provides a mechanistic description of how cortical D1R-sensitivity may critically influence WM maintenance during delay and manners in which altered sensitivity may harm the cognitive ability. The two striking features of D1R-sensitivity are its tight control over the level as well as size of optimal DA span facilitating optimum strength of sustained cortical activity during delay and the resulting impact on the robustness of sustained-firing activity against annihilation due to noisy perturbations. An important point to be noted is that the model takes into account only functional alterations in the dopaminergic synaptic transmission and does not consider anatomical alterations in the circuit’s connectivity.

### Significance and limitations of the neural mass model of the mesocortical dynamics

Highly detailed cortical network models [6, 7, 10, 71] are already available to elaborate the D1R-dependent dopaminergic modulation of cortical persistent activity during WM maintenance. These studies investigate the role of every minute component of the neuronal excitability as well as synaptic transmission in the network’s firing activity. By taking into account the empirical observations on the effect of D1R stimulation on these components [66–68, 70], the theoretical studies have laid down the fundamental picture of the biophysical driving forces behind the dopaminergic modulation in the PFC. However, the present issue with D1R-sensitivity naturally demands consideration of a more comprehensive prefronto-mesoprefrontal machinery, which involves the additional dynamics for regulating the cortical DA content in close association with the prefrontal activity. However, many quantitative intricacies of the dynamics within VTA, regulation of DA neurons activity by prefrontal cortex, involvement of tonic versus phasic activity of DA neurons and local cortical regulation of DA content are still sufficiently missing to construct an appreciably detailed network model of the mesocortical circuitry. Moreover, a detailed network model for this large a system would not only be cumbersome for computation but also be intractable for understanding its consequent dynamics.

Under such circumstances, a neural mass model of the mesocortical circuit may prove an effective framework. Such models conceptualize the bare essentials behind a system’s dynamics distilled out from its natural complexity [86, 87]and the details may be carefully amalgamated into minimal factors required for capturing the system’s original dynamics. Nonetheless, the physical quantities of interest in the present inquiry, viz. intensity of sustained-firing activity during delay and its robustness, are the functional features shaped at the population-level, instead of single independent neurons of the local network. Therefore, the mass model approach fits in well for addressing these issues. As a result, despite its simplicity and tractability, the model effectively captures salient attributes of the phenomenon of dopaminergic modulation as observed in the earlier experimental and theoretical studies.

The bifurcation profile observed in the earlier computational study by Brunel and Wang [7] (see Fig 10 of the study) exhibits loss of bistability at higher levels of D1R stimulation and, hence, is different from the bifurcation profile of cortical pyramidal activity obtained here with respect to the releasability parameter *R*_*DA*_ (Fig 2A). The former study considers an isolated cortical module and an independent parametric variation in the level of D1R stimulation. However, the level of D1R stimulation is not an independent parameter in the present study. Rather, it is shown to be strictly regulated by the extracellular cortical DA content, which is further governed by the cortical firing activity in a feed-back manner. Since the interactions at the mesocortical scale results into saturation in the cortical DA content with parametric increase in the *R*_*DA*_, there exists a limit to which stimulation of D1Rs can increase. Within this limit, the bistable bifurcation profile does not vanish. However, the nullcline analysis of the isolated cortical dynamics in the present dynamical model involves independent variation in D1R stimulation level (S1 Fig). Accordingly, the nullcline profile of the pyramidal activity obtained here indeed shows the loss of bistability at higher D1R stimulation, akin to the Brunel and Wang observation.

The earlier experimental studies on the dopaminergic modulation of cortical persistent activity have also applied controlled variation in the local cortical concentrations of D1R agonist and antagonist using iontophoretic techniques [11, 12, 14, 32]. This is similar to the parametric variation in the D1R stimulation in the earlier computational studies as well as the nullcline analysis for the pyramidal activity performed here. Consequently, loss of bistability at higher D1R stimulation has also remained profusely evident in the empirical observations. However, the present study suggests that such a bifurcation profile may not exist in vivo as there is a functional limitation on the rise of cortical D1R stimulation level under physiological conditions.

In reality, there exists multiple local populations of cortical neurons within a small region of the cortex such that each population is tuned to exhibit persistent activity for a specific feature or information of the stimulus presented in a WM task, such as spatial orientations in visuospatial WM tasks [39]. Therefore, there exists simultaneously multiple attractor states. However, while dealing with a single local population of cortical neurons, the present model projects a single attractor state with persistent activity. From the viewpoint of network’s dynamics, failures at the end of the delay period recorded for the behavioral performance of a subject may occur either through the premature collapse of the sustained-firing activity to the spontaneous-activity state or the transition of the firing state to another attractor during delay [84, 88, 89]. As far as the former route is concerned, the present study directly elaborates the ways in which the anomalies in dopaminergic modulation may affect WM-robustness and the behavioral performance. However, it also paves a way to explain the latter route to some extent. The observed increase in the shallowness of the basin of attraction associated with the sustained-firing state causing declined robustness under anomalous conditions of dopaminergic modulation is a biophysical property of the local cortical dynamics. Therefore, the various attractor states relying on the similar dynamical principles of sustained-firing activity would together get shallower and lesser robust under such conditions. In a way, the entire global potential landscape of sustained-firing attractors becomes shallower. Although the intensity of instability may not be identical for all the attractor states, it would be reasonable to envisage that transitions from one sustained-firing attractor to the other would become easier as well as frequent. Accordingly, the WM-robustness shown here in terms of PB and SNR is merely indicative of the WM performance.

Nonetheless, the ongoing discourse regarding the exact role of the sustained-firing activity as a neural correlate of WM maintenance in the PFC is worth considering. It is still under intense debate whether the sustained-firing activity itself stores relevant information about a presented stimulus [88, 89] or it serves as a top-down biasing control over other areas of the cortex, such as posterior parietal cortex (PPC) or inferior temporal cortex (ITC), to aid them in encoding the salient features in their local persistent activities [90, 91]. To some extent, it is quite apparent that the spatial location of the cue presentation in the visuospatial WM tasks is at least encoded by the PFC circuitry whereas other features of the visual stimulus have been suggested to be represented in the PPC, which normally responds towards these specific sensory stimulus besides WM tasks [89, 90]. Accordingly, complex visuospatial WM demands may simultaneously involve storage of information as well as top-down biasing by the sustained-firing activity in the PFC. However, the quality of sustained-firing activity in the PFC in terms of its mean firing frequency and robustness against noisy fluctuations is essential for the eventual behavioral performance of a subject undergoing WM task, regardless of which route it takes to shape the WM maintenance during delay. In fact, some studies have shown that robustness is a unique feature of PFC microcircuitry which avoids the loss of goal-directed memory in the presence of distractors whereas the other cortical areas lack this attribute [89]. Additionally, the robustness of persistent activity in the PFC has been observed as a requirement for the biasing control over the stable representations in PPC. Therefore, the observations made here are equally applicable to both the ways through which sustained-firing activity in the PFC may be involved in WM maintenance.

Another important fact is that, in the case of primates, DLPFC as well as medial prefrontal cortex (mPFC) both have been observed to show sustained-firing activity in WM tasks [3]. In this regard, domain-specific hypothesis proposed by Goldman-Rakic [38] suggests that spatial WM features are dealt by DLPFC whereas non-spatial features are dealt by mPFC. Contrastingly, a process-specific model by Petrides [92] hypothesizes that mPFC retrieves information from PPC and DLPFC does the job of monitoring the information. Although the present model involves only DLPFC, it is recognized that the present study is not limited to DLPFC but can also be applied to WM maintenance-associated persistent activity in mPFC. This is due to the fact that both these regions share similar cortical microcircuitry to some extent [37] and, thus, involve a common physical mechanism for the establishment of sustained-firing activity.

Notably, despite the overwhelming evidences for the role of cortical persistent activity in WM maintenance, there are ample empirical observations which also suggest that persistent activity during the entire span of delay is not necessary at all for the WM maintenance [2]. Across trials in a variety of WM tasks [80, 93, 94], it has been observed that a long initial span of the delay after the presentation of cue stimulus sometimes lacks a persistent activity and is rather characterized by a spontaneous activity state. Only in the response preparation phase of the delay, immediately before the response, the persistent activity rapidly appears and leads to a successful trial. This is indeed surprising, unless the spontaneous activity during delay itself stores the goal-directed information. A proposed dynamic coding model of WM maintenance [2] suggests that the heightened activity in a local cortical network at the instance of cue presentation can temporarily energize a hidden activity state of the network through short-term plasticity or coherence. This hidden state possesses a specific pattern of activity which can carry the desired WM information but generally stays in the network as an activity-silent state. At the instance of its cue-induced emergence, it can be transiently adopted by the spontaneous activity state of the network. Accordingly, this mode of WM maintenance during delay is referred to as the activity-silent WM maintenance [2]. It seems that the occurrence of activity-silent mode across several trials differs with the nature of WM tasks and depends particularly on the demand of parallel attention and processing [80]. However, the present model is not equipped with the essential framework to accommodate the activity-silent mode of WM maintenance during delay.

### Clinical implications in ageing and schizophrenia

The observed dependence of the various essential features of dopaminergic modulation on DA-releasability and D1R-sensitivity carries potential clinical implications. In the case of ageing, there occurs a substantial decrease in the cortical D1R-sensitivity [16, 17, 21]. Bäckman et al. [17] using PET study estimated a 14% average age-related loss of D1Rs BP per decade in DLPFC. In another PET study, Suhara et al. [21] using [11C]-SCH23390, a highly selective ligand for D1Rs, reported a 39% decrease in D1Rs BP in the frontal cortex with age. Keyser et al. [16] also observed a significant decrease in D1R density and reactivity of their high affinity sites in the frontal cortex with age. Interestingly, decrease in D1R-sensitivity is observed here to be associated with wider range of optimal DA content and relatively higher robustness of WM maintenance. However, these benefits are strongly counteracted by large shifts of the WM regime of cortical dynamics to higher DA levels. Here, with decrease in D1R-sensitivity, the associated optimal range of DA appears more and more unapproachable by the normal levels of DA-releasability of the mesocortical projections. The situation becomes more severe as the DA-releasability also exhibits a decline in ageing [16, 21, 36, 95]. Therefore, ageing may end up either in a complete loss of WM maintenance or a poor WM maintenance depending on the severity of D1Rs depletion as well as decline in the DA-releasability.

A contrary situation is witnessed in the case of schizophrenia where a chronic hypodopaminergic state of DLPFC leads to a substantial upregulation of cortical D1R density. PET studies by Abi-Dargham et al. [23, 24, 26] observed that [11C]NNC112 BP was significantly elevated in the DLPFC of unmedicated schizophrenic patients. A postmortem study performed by Knable et al. [22] also reported a significant increase in the BP of [3H]-SCH23390 in the prefrontal cortex of schizophrenic patients as compared to normal controls. Accordingly, it demonstrates the situation of elevated cortical D1R-sensitivity. It is observed here that high D1R-sensitivity causes the WM regime of cortical dynamics to shift to very low levels of DA. At first, it seems a homeostatic mechanism so that WM could be formed even under hypodopaminergic state, as has also been suggested earlier [23, 24, 26]. But this rescue doesn’t seem to be eventually much useful as the schizophrenic patients indeed show impairment of WM maintenance. The present observations suggest that too much responsiveness of cortical dynamics to even a slight change in cortical DA content makes it difficult to stay within the optimal range of DA under the conditions of natural fluctuations in the cortical DA content. This is aided by the fact that the optimal DA window also considerably shrinks with increase in D1R-sensitivity. Moreover, the associated WM-robustness also decreases under such conditions. Further, if there occurs an uncontrolled increase in DA content due to the administration of DA elevating drugs [11] or due to the heavy demand of a WM task [26], the cortical dynamics would easily shift to the very far sections of the post-peak region in the bifurcation profile, which may again lead to poor WM maintenance.

Currently, no well-defined protocol of medication exists for the cognitive deficit associated with DA-dysfunction [15, 96], owing to the limited knowledge of the several factors involved in the dopaminergic modulation of cortical activity. Yet, two genres of drugs are being examined for their medicinal potency: (a) drugs which are pharmacologically D1R agonists and antagonists [15, 20] (b) drugs which modulate the DA release probability of the afferent dopaminergic projections to cortex [25, 97]. The former has a direct role in regulating the cortical D1R stimulation whereas the latter does it indirectly via regulating the dopaminergic condition of cortex. Moreover, an efficient use of these drugs requires a trial-based estimation of the appropriate drug-combination and drug-dosage, which exhibit a huge unpredictable variability across the patients suffering from the same neuropsychiatric disorder [98].

It is shown here that one of the neglected aspects in the current clinical diagnosis, i.e. alteration in D1R-sensitivity, has a strong deterministic contribution to the otherwise unpredictable variability in response to DA-correcting drugs across patients. Features such as critical DA-releasability and cortical DA content required to capacitate cortical circuitry for WM function, modulation-associated DA window, the sharpness of the modulation profiles of neuronal activities, the optimal region of modulation and the associated optimal DA window, are significantly affected by alterations in D1R-sensitivity. This suggests that the drug-mediated tuning of cortical DA content to improve the cortical D1R stimulation based only on the knowledge of dopaminergic condition of the cortex is not sufficient. It should also be accompanied by the diagnosis of the intensity of alteration in D1R-sensitivity inflicted by the pathological condition. In fact, the precision of DA-tuning substantially varies according to the intensity of alteration in D1R-sensitivity and so is the effective drug-dosage [98].

Other clinically important aspects demonstrated here stem from the features of WM-robustness. It is shown that the optimal region of the modulation does not manifest only from the optimal levels of cortical sustained activity but also from the optimal levels of robustness during delay. Moreover, the effect of alteration in D1R-sensitivity on the robustness suggests that even if an optimal cortical sustained activity is achieved by retrieving an optimal cortical DA content, the associated robustness cannot be identically gained if the alteration in cortical D1R-sensitivity is not equally improved. This further indicates that there is no substitute of a remedy for altered D1R-sensitivity condition. The manner in which antipsychotics impact D1R-sensitivity [99] is unclear and therefore, its effect is not under appropriate clinical control. A perfect medication of cognitive deficits emanating from DA-dysfunction would necessarily require an amalgam of strategies which can together alleviate the anomalies in cortical DA content as well as D1R-sensitivity.

## Supporting information

S1 Fig The nullclines of the excitatory population activity, *a*_*PN*_, and the cortical D1R stimulation, *D*1*R*_*act*_. (A) For a given *D*1*R*_*sens*_, the solid black curve is the *a*_*PN*_-nullcline and the grey lines are the *D*1*R*_*act*_-nullclines for the different % values of DA-releasability, *R*_*DA*_, relative to *R*_*DA*_ = 0.0058*nM*.*ms*^−1^. As evident, increase in *R*_*DA*_ causes a rightward shift in the *D*1*R*_*act*_-nullcline. The point(s) at which a *D*1*R*_*act*_-nullcline for a given value of *R*_*DA*_ intersects the *a*_*PN*_-nullcline together defines the corresponding operating point(s) of the mesocortical system, where a point marked with solid circle represents the stable state and that marked with open circle represents the unstable state of the system. (B-C) As *D*1*R*_*sens*_ is increased, the rate of rightward shift in the *D*1*R*_*act*_-nullcline in response to variation in *R*_*DA*_ considerably increases, which illustrates a heightened response of the mesocortical system to variation in the cortical DA content.
(TIF)
