# Supplementary figures and images for: Prefronto-cortical dopamine D1 receptor sensitivity can critically influence working memory maintenance during delayed response tasks
Source: PLoS One. 2018 May 29;13(5):e0198136. doi: 10.1371/journal.pone.0198136 (PMC5973564; doi:10.1371/journal.pone.0198136)

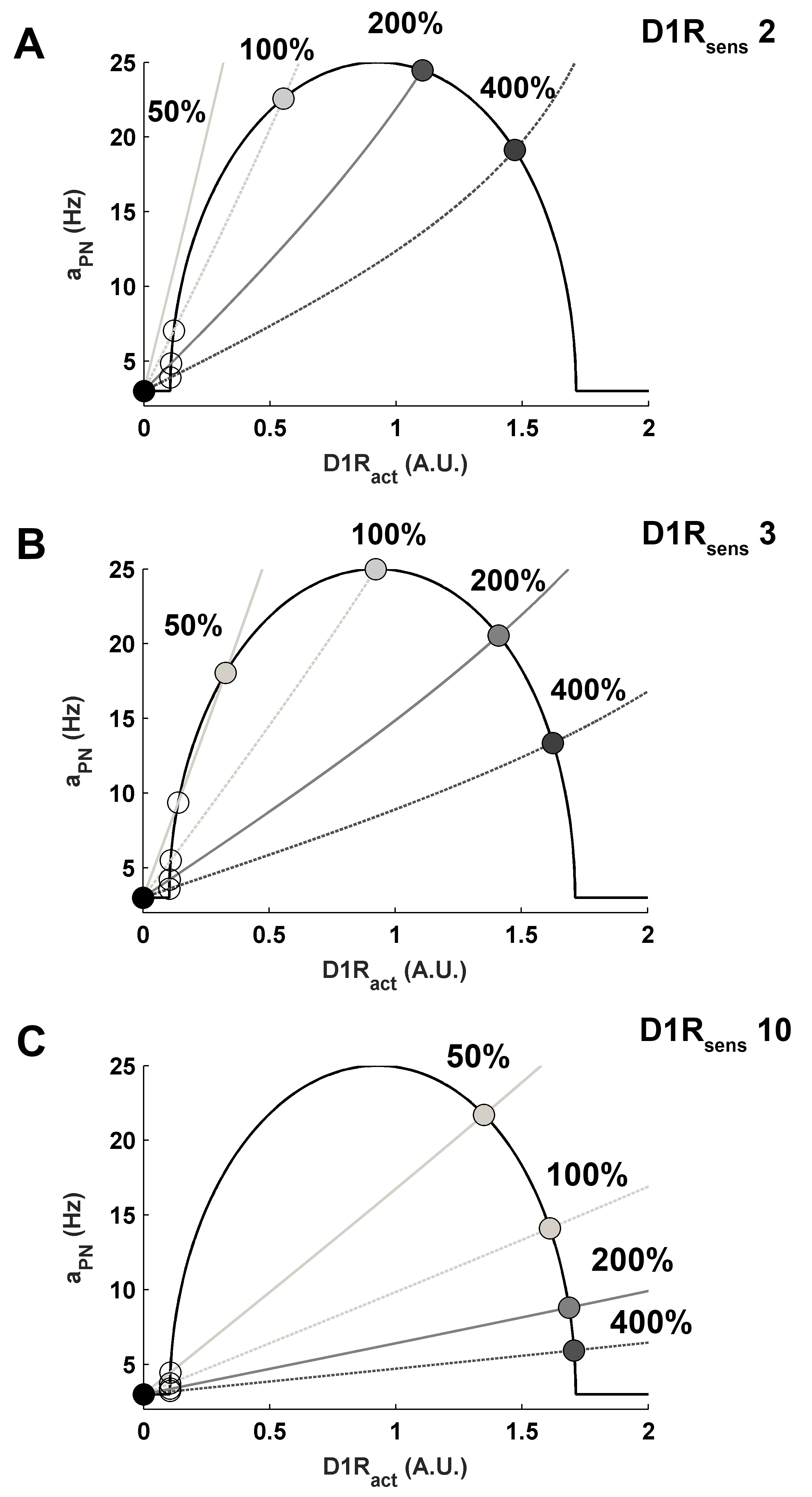

Supplement: S1 Fig — (A) For a given D1Rsens, the solid black curve is the aPN-nullcline and the grey lines are the D1Ract-nullclines for the different % values of DA-releasability, RDA, relative to RDA = 0.0058nM.ms−1. As evident, increase in RDA causes a rightward shift in the D1Ract-nullcline. The point(s) at which a D1Ract-nullcline for a given value of RDA intersects the aPN-nullcline together defines the corresponding operating point(s) of the mesocortical system, where a point marked with solid circle represents the stable state and that marked with open circle represents the unstable state of the system. (B-C) As D1Rsens is increased, the rate of rightward shift in the D1Ract-nullcline in response to variation in RDA considerably increases, which illustrates a heightened response of the mesocortical system to variation in the cortical DA content. (TIF) [file pone.0198136.s001.tif]
